# Employing Broadly Neutralizing Antibodies as a Human Immunodeficiency Virus Prophylactic & Therapeutic Application

**DOI:** 10.3389/fimmu.2021.697683

**Published:** 2021-07-20

**Authors:** Chengchao Ding, Darshit Patel, Yunjing Ma, Jamie F. S. Mann, Jianjun Wu, Yong Gao

**Affiliations:** ^1^ The First Affiliated Hospital of USTC, Division of Life Science and Medicine, University of Science and Technology of China, Hefei, China; ^2^ Department of Microbiology and Immunology, University of Western Ontario, London, ON, Canada; ^3^ Department of AIDS Research, Anhui Provincial Center for Disease Control and Prevention, Hefei, China

**Keywords:** HIV-1, AIDS, bNAb, prevention, therapy

## Abstract

Despite the discovery that the human immunodeficiency virus 1 (HIV-1) is the pathogen of acquired immunodeficiency syndrome (AIDS) in 1983, there is still no effective anti-HIV-1 vaccine. The major obstacle to the development of HIV-1 vaccine is the extreme diversity of viral genome sequences. Nonetheless, a number of broadly neutralizing antibodies (bNAbs) against HIV-1 have been made and identified in this area. Novel strategies based on using these bNAbs as an efficacious preventive and/or therapeutic intervention have been applied in clinical. In this review, we summarize the recent development of bNAbs and its application in HIV-1 acquisition prevention as well as discuss the innovative approaches being used to try to convey protection within individuals at risk and being treated for HIV-1 infection.

## Introduction

The causative agent of acquired immunodeficiency syndrome (AIDS) is human immunodeficiency virus 1 (HIV-1), a lentivirus from the *Retroviridae* family (1). HIV-1 predominantly infects CD4+ T cells which play a central role in the adaptive immune system by activating and modulating the activity of other immune cells (2). Individuals with AIDS experience a steady depletion in CD4+ T cells, rendering them severely immunocompromised (3) and susceptible to opportunistic infections (4). Since the first published report of AIDS, in June 1981 (5), 35 million people have died from AIDS-related illnesses. Despite educational and preventative measures, approximately 38 million people are currently living with HIV-1 and 1.7 million new infections are reported in 2019 (http://www.unaids.org/en). The disease has a disproportionate prevalence with a major dominance in Africa, where it has dramatically decreased life expectancy and economic growth (3, 6, 7). Nevertheless, with thorough scientific investigation, several breakthroughs have been made to alleviate the lethality and devastating impact this disease has had on communities.The development of combination anti-retroviral therapy (cART) was a crucial event in decreasing HIV-1-associated morbidity and mortality (8–10). The efficacy of ART is characterized by its ability to inhibit various aspects of the HIV-1 replication cycle and thereby sustain viral loads below the limits of detection. Consequently, effective viral suppression by ART will improve the immune function, reduce AIDS-related complications, and improve the overall quality of life (10–12). More importantly, HIV-1 acquisition has transformed from a highly lethal infection to a life-long, treatment-manageable affliction. Despite these positive implications, ART can’t cure the disease due to the existing of HIV-1 latent reservoir. Moreover, it may raise significant concerns on the sustainability and affordability of ART and create a potential for global economic issues to enforce HIV-1 patients to a lifelong dependency on ART (13). Neutralizing antibodies (NAbs) therapy or effective induction of its production as one of the most promising alternative method have received extensive attention. In this review, we will discuss the targets of antibody responses on HIV-1 envelope (Env), the generation of such antibodies, and the progress and viability of current HIV-1 prophylactic and therapeutic methodologies, with a major focus on leveraging bNAbs for HIV-1 prophylactic and therapeutic applications.

## HIV-1 Envelope

### HIV-1 Envelope, Diversity and the Obstacles for Anti-HIV-1 Vaccine Development

It is widely believed that the holy grail of HIV-1 prophylactic vaccine development is an Env-based immunogen that elicits broad immune response against a wide array of HIV-1 strains. However, the HIV-1 Env glycoprotein has several special characteristics that render the virus evading the attack from host immune response. The HIV-1 genome consists of two copies of single stranded RNA surrounded by a capsid and by a viral membrane. Situated on the outer membrane are Env glycoproteins that are incorporated into the virions as they bud from the host cell. The Env glycoprotein of HIV-1 is synthesized in the rough endoplasmic reticulum and processed into gp120 and gp41 *via* cleavage by host protease furin (14, 15). Surface gp120 and the transmembrane gp41, associated noncovalently, are determinants of viral tropism and are involved in the promotion of viral and host cellular membrane fusion by recognizing and interacting with particular receptors (16). Specifically, Env gp120 serves as the receptor-binding component and engages CD4 and a coreceptor (CCR5 or CXCR4), while gp41 serves as a means for viral core entry into the host cell. The successful binding and entry of HIV-1 involves several conformational changes that expose conserved regions of the virus and typically occurs in a two-step process outlined in detail by Wilen et al. (17). Briefly, CD4 binding to the constitutively accessible CD4 binding site (CD4bs) on gp120 induces a conformational change, which triggers both high affinity CD4 binding and structural rearrangement of the Env trimer to reveal the chemokine-binding site. The exposure of chemokine-binding site promotes further engagement by gp120 and induces another conformational rearrangement of the Env trimer, triggering gp41 activation. The consequent refolding of gp41, as a result of its activation, induces viral and target cell membrane fusion and subsequent deposition of the viral core within the target cell (17). Env proteins are amongst the most immunogenic components of HIV-1 particles as they are accessible targets being expressed on the viral membrane (18, 19).

Although the ability of HIV-1 to rapidly mutate its genome enhances antigenic variation of surface glycoproteins, the domains that bind to CD4 and co-receptor are relatively conserved. gp120 possesses five conserved regions (C1-C5) that are interspersed between five regions of considerable sequence variability, often called hypervariable loops (V1-V5) (20). The variable regions occlude the constant regions, thus antibodies are primarily raised against variable regions rather than constant regions (20).

The extreme genetic diversity of the virus results from its highly error-prone reverse transcriptase, a high tendency for recombination driven by the constant evolutionary pressure of avoiding detection and destruction by the immune system, and an extremely rapid turnover *in vivo* (21, 22). Currently, there are four distinct groups of HIV-1: M, N, O and P. Group M is further subdivided into nine distinct subtypes (A-D, F-H, J, and K) and numerous additional circulating recombinant forms (CRFs) (21, 23). Some of these CRFs have recombined with other subtypes or other CRFs to form “second generation” recombinants (9). Genetic inter-clade diversity ranges from 20-30%, diversity within clades reaches as high as 12%, and circulating viruses can differ within the highly variable Env protein by up to 38% (22, 24). As a consequence, this vast diversity of the virus makes it very difficult to design an immunogen that can account for all existing variants of HIV-1.

Moreover, HIV-1 utilizes host-derived, non-immunogenic glycans to mask its Env to evade immune recognition (25–27). gp120 contains approximately 25 N-glycosylation sites that form a glycan shield, which serves as another mechanism to overcome immune defenses (20). Nearly 50% of Env’s molecular mass is composed of host derived N-linked sugars, and such carbohydrate moieties prevent immune recognition by occluding highly immunogenic epitopes on gp120 (18, 28). The role of Env glycans in attenuating recognition by immune cells has been demonstrated in several studies where removal of specific glycans in V loops, within C2-C4 region, and gp41 exposed relevant bNAb epitopes (29–31). In one such study, rhesus monkeys infected with Simian Immunodeficiency Virus (SIV) mutants lacking glycosylation in the V1 region of gp120 resulted in the production of higher neutralizing antibody titers than those infected with wild type SIV (32). Specific to the recessed pocket on gp120 containing the CD4bs, removing glycans peripheral to it increases the sensitivity of the virus to neutralization (33–35).

### Types of Humoral Response Against Env

**Figure 1:**
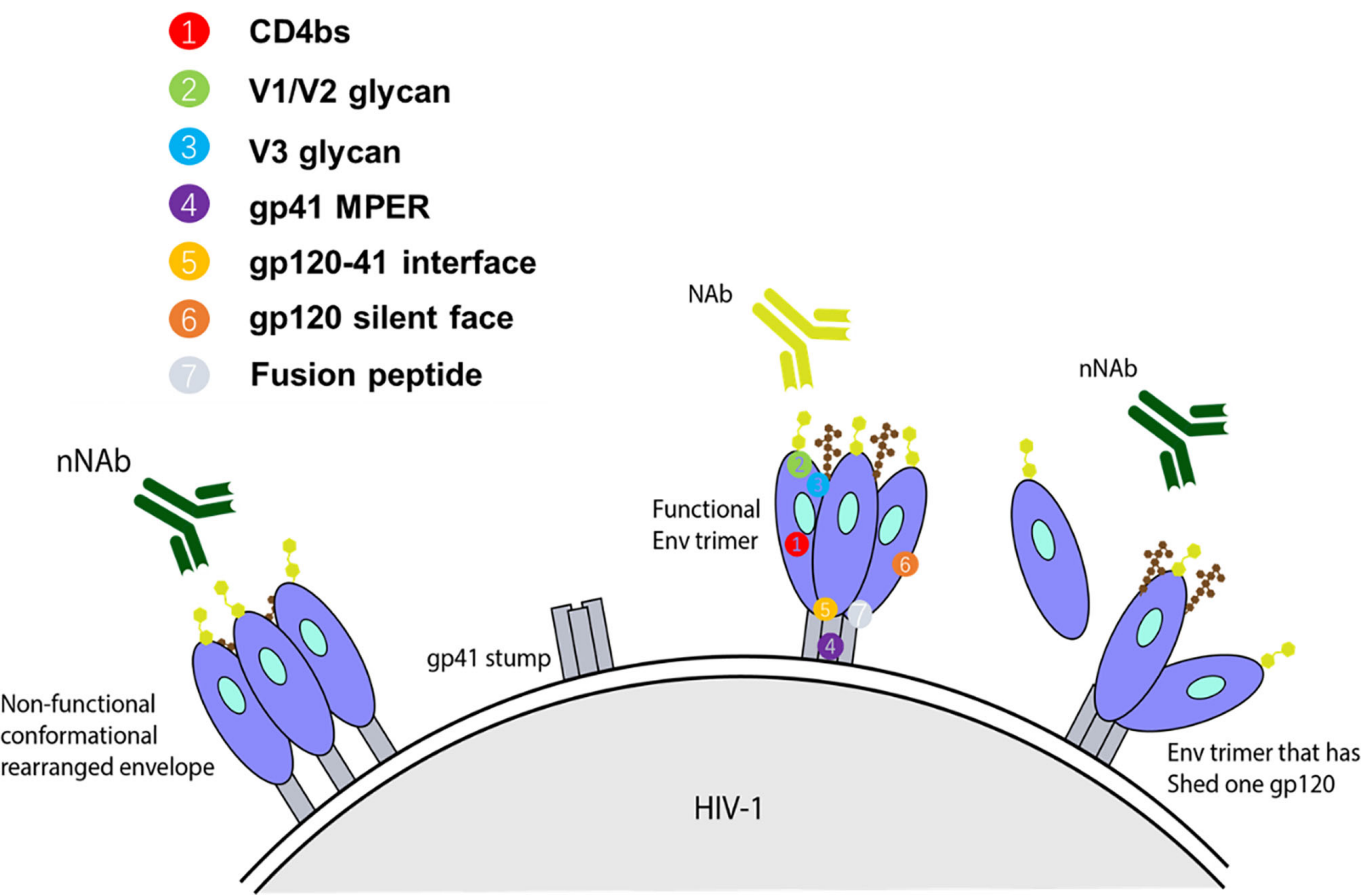
Representation of some forms of Env targets present on infecting HIV-1 strain and available to elicit their respective class of antibody response. For an anti-HIV-1 antibody to have neutralizing activity, it should interact with functional spikes on Env trimers that mediate HIV-1 entry into target cells (38, 39). Non-neutralizing antibodies typically target Env epitopes absent from the functional spikes of the native Env trimer (39, 40). The targets for autologous nAbs are V loops and other regions of gp120 with relatively high sequence variation on functional spikes between strains, and therefore, they can only bind Env trimers from the infecting strain. Heterologous bNAbs on the other hand typically target the relatively conserved regions, based on sequence or amino acid homology, with some targeting the variable loop. Collectively, the known spectrum of bNAb targets encompass the CD4bs, glycan dependent epitopes V1/V2 and near the base of V3/C3, linear epitopes in the membrane proximal external region (MPER) of gp41, gp120-41 interface, gp120 silent face, and fusion peptide.

It has been reported that bNAbs neutralizing heterologous viruses of diverse subtypes could be developed during chronic infection in a small portion of HIV-1 infected individuals (36, 37). The resultant antibodies can be classified into three groups based on their ability to target and neutralize a range of HIV-1 strains (**Figure 1**) (41). The first group of antibodies involves a class of antibodies that are unable to neutralize viruses, including those that have Env sequences identical to immunizing antigen. However, it is still likely that such non-neutralizing Ab (nNAb) can perform antiviral function through Fc-mediated activities (41). Antibodies bound to the Env proteins can attach to FcγR expressed on innate immune cells and trigger Ab-dependent cellular cytotoxicity (ADCC) and Ab-mediated cellular phagocytosis (ADCP). ADCC is mainly mediated by natural killer (NK) effector cells, whereas monocytes, macrophages or dendritic cells that can internalize Ab-bound virus or Ab-coated cells are responsible for ADCP (42). These two Fc-mediated processes are dependent on IgG subclass and Fc glycosylation, with some mutations of the Fc region, such as the removal of the Fc glycan fucose residues, increasing the effectivity of ADCC (41).

The second group of antibody response encompasses antibodies that neutralize virus but in a highly strain-specific, autologous manner. Often, these antibodies exert selective pressures that drive viral resistance to the humoral response. Consequently, they promote survival of escape variants that have been generated *via* insertion/deletion mutations in gp120 variable regions through amino acid substitutions and changing surface glycosylation sites (18, 19, 43, 44).

The third group of antibodies is associated with a response able to neutralize a wide array of HIV-1 strains and is therefore termed bNAbs. *In vitro*, bNAbs have been confirmed to neutralize the majority of HIV-1 strains with a half maximal inhibitory concentration (IC_50_) of less than 1 μg/ml (41, 45). The known spectrum of bNAb targets encompass the CD4bs (46, 47), glycan dependent epitopes V1/V2 and base of V3/C3 (48–51), linear epitopes in the membrane proximal external region (MPER) of gp41 (52), gp120-41 interface (53, 54), gp120 silent face (55, 56), and fusion peptide (57) (**Figure 1**). The ability of bNAbs to target conserved regions derives from years of continuous affinity maturation. This endows them with mechanisms to cope with the variability that surrounds the small regions of conserved Env trimer (41).

## Anti-HIV-1 Antibodies

### First Generation bNAbs

During chronic HIV-1 infection, approximately up to 50% of infected individuals produce high levels of neutralizing antibodies (NAbs) against Env (36, 58, 59). Among them, approximately 1% are “elite neutralizers” and produce bNAbs, typically after 2-4 years (60). The first-generation anti-HIV-1 bNAbs were discovered when researchers found antibodies capable of neutralizing different HIV-1 subtypes in the early 1990s. These bNAbs were isolated by using phage display or human hybridoma electrofusion. The first generation bNAbs include b12 (CD4bs), 2G12 (viral glycan), 2F5 and 4E10 (gp41 MPER) (**Table 1**) (71). 2G12 bNAb recognizes α1→2 mannose residues proximal to V3 and V4 loops on gp120 (116). The epitope recognized by 2G12 is conformationally sensitive, strongly depending on asparagine glycosylation in the C2-, C3-, C4-domains, and the V4 loop (20). 2F5 and 4E10 possess a unique ability to bind strongly to the MPER region with one Fab fragment, while having a low affinity for an alternative target on Env with the other Fab fragment (72). This mode of heterogenous binding seems to increase the neutralization efficacy of primary HIV-1 isolates.

**Table 1:** Categories, efficacies, and research development of broadly neutralizing HIV-1 antibodies.

Traget site (see **Figure 1**)	Antibody designation	Potency* (Median IC50,μg/mL)	Breadth (% of n pseudoviruses, IC50<50μg/mL)	Similar monoclonal antibodies	Year generation	References
CD4bs	VRC01	0.25	91	VRC02, VRC03, VRC232	2010	(61)
				NIH45-46, 3BNC60, BNC62, 3BNC117, 12A12, 12A21, 12A30, VRC-PG04,VRC-CH31		
	HJ16	1.16	36	?	2010	(62)
	b12	1.79	40	?	1991	(63)
	CH103	4.54	55	CH104 to 106	2013	(64)
	N6	0.04	98	?	2016	(65)
V1/V2 loop	PG9	0.109	78	PG16, CH101	2009	(50)
	PGT145	0.3	78	PGT141 to 144	2011	(66)
	2G12	1.45	28-39	?	1994	(67)
V3 loop	PGT121	0.07	70	PGT122, PGT123	2011	(49)
	10-1074	0.036	66	?	2012	(49)
gp41 MPER	2F5	1.44	67	m66	1992	(68)
	4E10	1.303	98	?	1994	(69)
	10E8	0.222	98	7H6	2012	(52)
gp120-41 interface	8ANC195	0.415	66	8ANC3040, 8ANC3484	2014	(53)
	35022	0.033	62	?	2014	(54)
gp120 silent face	VRC-PG05	0.8	27	VRC-PG04	2018	(56)
	SF12	0.2	62	SF5	2019	(55)
Fusion peptide	ACS202	0.142	45	PGT151	2017	(70)
	VRC34.01	0.3599	49	PGT151	2016	(57)

*For neutralization potency, the geometric mean value among neutralized viruses is shown. TZM-bl/pseudovirus neutralization assay was used to evaluate the neutralization potencies and breadths of the antibodies.

?, unknown.

Studies carried out with HIV-1 pseudoviruses of different subtypes have demonstrated that first-generation bNAbs exhibit low to moderate breadth and neutralization potencies (20). Therefore, achieving the desired efficacy as a therapeutic requires high concentrations of these bNAbs, with an inherent limitation to the range of HIV-1 isolates that can be neutralized. Additional undesirable characteristics of first generations bNAbs that impede their therapeutic applications have also been outlined. Firstly, 2G12 has a unique structure where each light chain (LC) is bound to the constant region of one heavy chain (HC) and variable region of the other heavy chain, resulting in the cross-association of the HCs, and consequently the Fab-fragments are also unusually closely aligned (20). Moreover, 2F5 and 4E10 are self-reactive and b12 is a phage-derived Fab-fragment Ab generated by random pairing of heavy and light chains that may have never existed in nature (71).

### Second Generation bNAbs

The discovery of high throughput single-cell B-cell receptor(BCR) amplification and novel soluble Env selection tools, together with the ability to culture memory B cells, has permitted identification and isolation of second generation bNAbs that are more potent and have a broader coverage (38, 50). Within these, antibodies that bind to the CD4bs are among the broadest, reaching coverage of up to 98% against cross clade viruses (73). The classification of CDbs bNAbs is divided into two major types, complementarity-determining region 3 (CDRH3) dominated and VH-gene restricted, based on their ontogeny and mode of recognition. CDRH3 dominated bNAbs make contact with their target site on the Env primarily through their CDRH3 regions and can be subdivided into four classes: CH103, HJ16, VRC13, and VRC16 classes; whereas VH-gene restricted CD4bs bNAbs make contact primarily through their CDRH2 regions and are subdivided into two classes: VRC01-class antibodies and 8ANC131-class antibodies (74). VRC01, VRC02, and VRC03 were isolated in 2010 from an HIV-1-infected individual living with untreated infection for over 15 years (61). These three bNAbs are highly somatically mutated somatic variants. While VRC01 and VRC02 are somatic variants of the same IgG1 clone with identical CDRH3 regions, VRC03 likely originated from a different IgG1 clone although derived from the same heavy chain alleles as VRC01 and VRC02 (61). It has been demonstrated that VRC01 can neutralize 91% of pseudovirions (of 190 Env-pseudotyped viral strains representing all major clades and circulating recombinants) at an IC50 of <50 μg/mL, and neutralize 72% of primary isolates at an IC50 of <1 μg/mL (38, 61). VRC02 exhibited similar properties as VRC01, however VRC03 was much less broad than VRC01 and VRC02, neutralizing 57% of the pseudovirions at an IC50 of <50 μg/mL.

Following the discovery of VRC01, numerous other CD4bs-targeting antibodies were identified, including NIH45-46, 3BNC117, and 12A12. NIH45-46 is a more potent clonal variant of VRC01 with high sequence and structural similarities to VRC01, yet it has a distinct mode of binding to gp120 (47). An amino acid substitution from glycine (G) to tryptophan (W) at position 54 yielded NIH45-46^G54W^ (47). This mutation increased the interactive surface between NIH45-46^G54W^ and gp120, which resulted in enhanced potency and breadth (47). For many years since its discovery in 2011, 3BNC117 had the greatest potency and breadth of all the CD4bs-targeting second generation bNAbs; the majority of tested viruses were more sensitive to 3BNC117 than VRC01 (74). 3BNC117 has been tested in phase IIa clinical trial to verify its safety and potential in suppressing viral rebound during ART treatment interruption (75). Recently discovered monoclonal CD4bs antibody, N6, is the most potent bNAb described thus far, and has one of the highest neutralization breadths. In a study involving 181 pseudoviruses from various clades, N6 was able to neutralize 96% of viruses at a median IC50 of 0.038 μg/ml. Focusing on clade C pseudoviruses, N6 neutralized 98% of 171 at a median IC50 of 0.066 μg/ml. N6 has a unique mode of Env recognition, including its ability to avoid steric clashes with the highly glycosylated V5 region as well as ability to tolerate loss of antibody contacts with the CD4bs or V5 region (65, 73). These properties enable N6 to overcome major mechanisms of resistance, thus it is able to neutralize many isolates that were resistant to VRC01 and other CD4bs antibodies (73).

Somatic variants PG9 and PG16, discovered in 2009, were among the first bNAbs identified to target the gp120 V1/V2 loops. Characteristic features of antibodies targeting the V1/V2 loops include exceptionally long CDRH3 arms to penetrate through the gp120 glycan shield to access the protein surface beneath (20). PG9 and PG16 possess notable neutralization breadth and potency, capable of neutralizing 78% and 73% of pseudoviruses, respectively (50). CAP256 was isolated from an HIV-1 subtype C-infected individual and has a tendency to neutralize subtype A and C viruses (76). CAP256 has a long CDRH3 loop that binds to a quaternary epitope within V1/V2 region, however it is highly specific for residues 159-171 in the V2 loop that make up the FN/LRD-K-K motif (76). PGDM1400, isolated in 2014, also interacts with the gp120 V1/V2 quaternary epitope and exhibited high breadth (neutralized 83% of viruses in a cross-clade 106-virus panel) and remarkable potency (median IC_50_ of 0.003 μg/mL) (77).

A number of broad and potent bNAbs that target the V3 loop region have been described, including PGT121, PGT128, PGT135, 10-1074, and AIIMS-P01. The high-mannose glycans on N332 are commonly targeted by these bNAbs, and they also possess long CDRH3 loops to penetrate the gp120 glycan shield (20). PGT121 utilizes a unique mode of action of neutralization by inhibiting CD4 binding to gp120 through allosteric mechanisms (78). It is postulated that allosteric interactions between PGT121 and key structural elements within V3 locks gp120 into a conformation that impedes CD4 binding. It is worth noting that PGT121 and 10-1074, in contrast to the other V3 loop-targeting bNAbs, bind to complex-type rather than high-mannose glycans on gp120 (49). AIIMS-P01 was recently isolated from a clade C chronically infected pediatric elite neutralizer. It is an HIV-1 N332 supersite-dependent bNAb and can neutralize 67% of HIV-1 cross-clade viruses (79).

The fourth major site of vulnerability that bNAbs can target is gp41 MPER. 10E8 is an MPER-specific antibody that demonstrated ability to neutralize 98% of tested pseudovirions at an IC_50_ of <50 μg/mL (52). 10E8 possesses a 22-amino-acid long CDRH3 loop that makes contacts with highly conserved hydrophobic gp41 residues, along with a narrow band of residues from CDRH1 and CDRH2 (52). Unlike other MPER-targeting antibodies, 10E8 is neither polyreactive nor lipid-binding (52). In recent years, three other bNAbs targeting sites, including gp120-41 interface, gp120 silent face and fusion peptide, were discovered. The bNAbs, relevant targeting sites, neutralization potencies and breadths were showed in **Figure 1**.

### Role of Non-Neutralizing Antibodies (nNAbs)

Neutralizing Abs generally bind to epitopes on functional trimeric Env and prevent subsequent virus-cell engagement, ultimately preventing infection. On the other hand, nNAbs commonly bind non-functional conformations and epitopes absent from functional Env spikes. Such conformations include open Env trimers, gp140 monomers, and cell receptor engagement-induced gp41 stumps (**Figure 1**). In several studies utilizing humanized mice, it has been demonstrated that nNAbs can provide protection through its Fc region (involved in the mediation of ADCC and ADCP), while also placing selective pressure and evolutionary constraints on the viruses (80, 81). In one such *in vivo* study, Horwitz et al. demonstrated that although nNAbs are less efficacious than bNAbs, they can provide modest protection against and change the progress of HIV-1 infection. To show this, a replication competent HIV-1 indicator strain (HIVivoHA) was generated with the ability to express HA-tag on the surface of virions and was used to infect cells. *In vitro*, the anti-HA antibodies were able to bind but not neutralize HIVivoHA. However, when challenged with HIVivoHA *in vivo*, the anti-HA antibodies protected against HIV-1 infection, reduced viral load in established infection, and cleared virus-infected cells. Similar results were obtained with passive transfusion of 246D, an anti-gp41 nNAb, when challenged with tier 2 HIV-1 viruses (HIV-YU2 or HIV-YU2-infected cells) (80). Contrary to these findings, some macaque studies suggest that nNAbs may reduce the number of founder variants, however, they do not protect against infection (82, 83). The conflicting results point to the lack of clarity of the sufficiency of Fc associated protection by nNAb against HIV-1 infection. Besides, nNAbs segment Fc also mediates antibody-dependent cell-mediated virus inhibition (ADCVI) and antibody-dependent complement–dependent cytotoxicity (ADCDC) *via* binding to the corresponding Fc receptors (Fcγs) on the surface of effector monocytes, macrophages, dendritic cells, or natural killer cells.

In a recent study, Anand et al. showed that two ADCC-mediating antibodies, anti-coreceptor binding site (CoRBS) and anti-cluster A antibodies, preferentially bind to the open conformation of Env glycoprotein (84). The binding of anti-CoRBS Abs resulted in the further open conformation of Env, facilitating anti-cluster A antibodies interact with the protein. They found that it is required that both antibodies bind to the same gp120 for the subsequent interaction with soluble dimeric FcγRIIIa. Furthermore, Fc regions of the two Abs are required to mediate robust ADCC, indicating that they act in a sequential and synergistic fashion.

## Generation of bNAbs

### Possible Mechanisms for Generation of bNAbs

B cell maturation is one of the proposed mechanisms for the humoral system developing bNAbs. The B cell repertoire consists of follicular (FO), marginal zone (MZ), plasmablast and plasma cells, as well as memory B cells. FO B cells, which are mature but inactive, are the most common type of B cells (85). They have a distinct phenotype from other B cell populations, recirculate in the blood, and can become either antibody-producing plasma or memory B cells with help from cognate T cells. They are responsible for generating the majority of high-affinity antibodies during an infection. MZ B cells are found mainly in the marginal zone of the spleen and lymph nodes and serve as the first line of defense against blood-borne pathogens (86). They preferentially undergo T cell independent activation, but can go through T cell dependent activation as well (85). Memory B cells are dormant B cells that arise from B cell differentiation following prior antigen recognition and T cell help (87). During chronic presence of HIV-1 specific antigens, these memory B cells go through multiple rounds of affinity maturation which allows for the gradual recognition of viral variants that emerge over time, and leads to the potential production of bNAbs (43, 88–90).

Germinal centers (GC) are the conveyors of affinity maturation. GCs consist of antigen-specific B cells that undergo proliferation and multiple rounds of somatic hypermutation (SHM) of the BCR in the dark zone, and subsequent selection in the light zone. Here, a fraction of the selected B cells returns to the dark zone for additional proliferation and rounds of mutations (91). Mutations in the BCR can alter its affinity towards its specific antigen. B cells possessing a high affinity BCR will capture more antigens and consequently express more antigen derived peptide on its MHC-II, and thus receive more help from follicular helper T cells (Tfh cells) (39, 40). Tfh cell help involves many molecules including CD40L, IL-21, IL-4, CXCL13, and SLAM. In one mouse study, one of two populations of GC B cells with the same BCR was given additional Tfh help. The population of GC B cells that received extra Tfh help showed increased clonal expansion. Collectively, help provided by Tfh cells contribute to the survival, proliferation, somatic hypermutation, and isotype class switching of GC B cells, all of which combine to select for high affinity B cells clones by Tfh cells (39). GC Tfh cells are not only necessary but also limiting in the production of HIV-1 bNAbs. In a human cohort, using a combination of Tfh cell surface markers to track Tfh cells, the authors demonstrated a correlation between frequency of memory Tfh cells and anti-HIV-1 bNAb generation (92). Similarly, at the primate level, SIV and Simian-Human Immunodeficiency Virus (SHIV) challenge studies in rhesus macaques have also shown an association between Tfh cells and generation of NAb breadth. Additionally, rhesus monkeys with the highest frequency of GC Tfh cells developed the highest SIV Env-specific antibody titers (93). Another study postulates that increasing the magnitude of HIV-1-specific Tfh response, or the breadth of the Tfh repertoire, can facilitate the evolution of anti-HIV-1 bNAbs (94).

The key players and complex interplay of genetic factors predisposing to bNAb development are still being investigated, however, predisposition to natural control of HIV-1, evident in elite neutralizers, involves HLA-I alleles that allow for a robust CD8+ T cell response (95). Inherently, characteristics of the immune system actually restrict the development of bNAbs. The relatively long CDRH3 typical of bNAbs, are generated at the stage of V(D)J recombination, and therefore restricting the human immunoglobulin repertoire in the variety of specific V(D)J rearrangements encoding germline precursors of HIV-1 bNAbs. This in turn explains the rarity of their presence. Conversely, the accumulation of a large number of mutations, generated by somatic hypermutation during multiple cycles of affinity maturations are also difficult to sustain (90).

The involvement of B-cell dysfunction is also a potential topic of study in the development of bNAbs. As a collateral from virus replication and mass-immune activation in HIV-1 infection, the B cell compartment undergoes profound dysregulation. Indicators of B cell dysregulation involve the delayed antibody response in acute infection, an increased activated memory B cell and exhausted B cell subpopulations, polyclonal immunoglobulin production through non-specific activation of plasmablasts, and reduced population of long-lived plasma cells (96–101). B cell dysregulation may be attributed to the transport of Nef to B cells *via* infected macrophages. Introduction of Nef to B cell may alter class-switching and germinal center responses (102). An alternate but equally likely cause of B cell dysregulation may be the early cytokine storm associated with HIV-1 infection and constitutive dysregulation caused by infection and depletion of T cells (103). Despite the effects of B cell dysfunction, it does not prevent the generation of bNAbs which requires further investigation. No correlation has been observed between the extent of dysfunction of circulating B cells during chronic infection and breadth of neutralization (37, 104).

It has been shown that the development of anti-HIV-1 bNAbs is associated with high genetic diversity of HIV-1 Env glycoprotein and its evolution. Mabvakure et al. compared the evolution of Env in eight HIV-1 patients developing bNAbs with six donors with similar viral loads but without bNAb developed over three years of infection (105). They found that overall evolutionary rates ranged from 9.92 x 10^(-3)^ to 4.1 x 10^(-2)^ substitutions/site/year and viral diversity from 1.1% to 6.5% across Env, and there was no significant difference between bNAb donors and non-bNAb donors. Interestingly, in the targeted epitopes, patients with bNAb had more positively selected residues than those without bNAbs, and the selection pressure increased at these residues along with the onset of breadth. These data indicate that the induction of HIV-1 bNAbs is most likely resulting from the highly directed evolution for virus.

Simonich et al. described the bNAb evolution in an infant for the first time. In the BF520.1 V3-glycan directed lineage of this infant, there was evolution of heterologous cross-clade neutralizing activity within 6 months of infection. Interestingly, to achieve the full breadth of the mature antibody, they found that only 2% SHM was needed, and that the features of pathway for this infant antibody development were distinct from adult antibodies, which may be amenable to better vaccine responses (106).

### Eliciting a bNAb Response Using Immunogens

**Figure 2:**
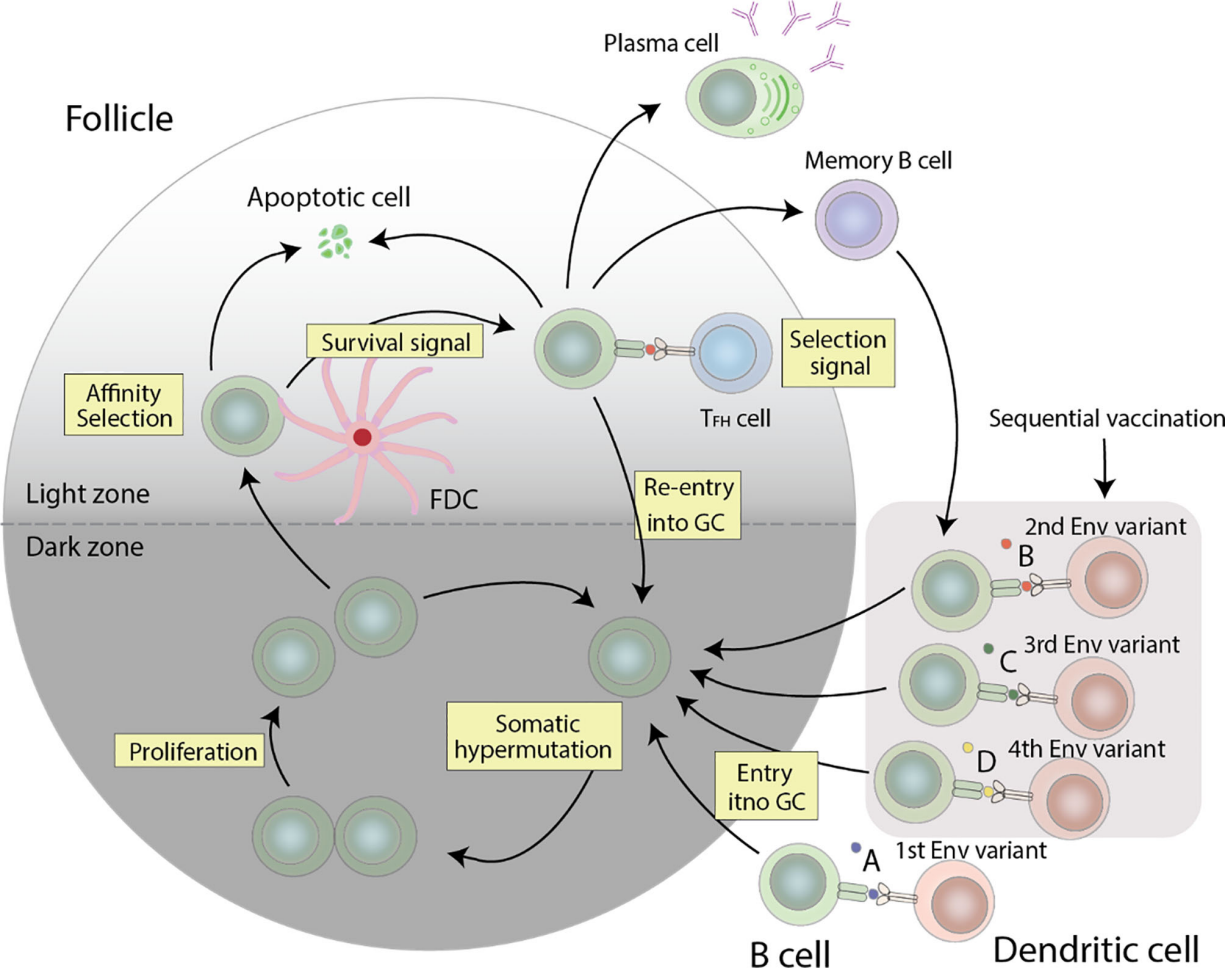
Germinal center reactions that can lead to the production of anti-HIV-1 bNAbs. HIV-1 specific B cells undergo proliferation and somatic hypermutation of the BCR in the dark zone, followed by affinity selection in the light zone. The resulting cells compete for the limited Tfh cell help, and cells with a high affinity BCR get more help than those with a low affinity BCR, which consequently undergo apoptosis. The surviving cells can either differentiate into plasma cells, re-enter the GC for additional rounds of somatic hypermutation and proliferation, or differentiate into memory cells. Re-entry of these memory cells with stimulation from other Env variants will allow for further affinity maturation. The combination of these events forms a cycle needed to generate anti-HIV-1 bNAbs.

In an attempt to effectively elicit a broad humoral immune response, various vaccine strategies have been explored, including designing of consensus or ancestral proteins (107–110), modifying Env variable regions or glycosylation sites (107, 110), and replicating Env-CD4 fusion transient intermediates (110, 111). To overcome HIV-1 diversity in particular, a frequently used method is the sequential immunization strategy. This strategy revolves around the idea that by sequentially exposing the host to a collection of Env variants representing the viral quasispecies members isolated from an individual that developed bNAbs, the host is able to undergo a virtually slightly different humoral response. Diversification of the Env gene would drive antibody maturation by presenting new epitopes in diversified variants and focus the humoral response on more conserved epitopes (110) (**Figure 2**).

Alternatively, a novel vaccine priming strategy that emphasizes germline as the target and initiates the affinity and maturation of specific germline precursor B cells has been of great interest. This strategy allows for immunofocusing by preferentially activating bNAb-precursor B cells, subsequently allowing for productive bNAb-like somatic mutations, and ultimately resulting in the production of memory B cells that can be boosted with Env immunogens to select for additional productive mutations (8, 112, 113). In some cases, inferred germline (iGL) precursors of bNAb, particularly ones involving the V1/V2 loops at the apex of the trimer, have high affinity for native Env from particular HIV-1 isolates (114, 115), simplifying the design of priming immunogens to be derived from Env of these isolates (77, 116). For other iGL bNAb precursors, no such wild-type Env has been found to bind with high affinity (113, 117), thus requiring the design of modified Env as the priming immunogen. An example of such an immunogen is the eOD-GT8 molecule which has been designed and selected to bind with varying affinity to various iGL versions of VRC01-like bNAbs (118). The efficacy of designed priming immunogens has been evaluated in immunization experiments in mouse models that have been engineered to have the precursor form of VRC01 IgH or IgL genes integrated into the corresponding mouse Ig loci. Using these “knock-in” mice, a single eOD-GT8 immunization was demonstrated to stimulate the production of a VRC01-class memory response in at least 29% of the immunized mice despite the presence of a low frequency of VRC01-class precursor per mouse (118). It is expected that after the priming of germline precursor, a potent induction of bNAbs will require sequential boosts, driving a succession of germinal-center reactions to select sufficient mutations.

In a recent study, by using neutralization data from four large virus panels, Bricault et al. mapped viral signatures comprehensively with bNAb sensitivity, including amino acids, hypervariable region characteristics, and clade effects across four different classes of bNAbs (119). They then employed the bNAb signatures (defined for the V2 epitope region of HIV-1 Env) to design a signature-based epitope targeted (SET) vaccine. A trivalent vaccine (V2-SET) was created by introducing V2 bNAb signature-guided mutations into Env 459C, and was used to immunize guinea pigs. The results showed that V2-SET vaccines elicited broader NAb responses when compared with Env 459C alone, indicating that bNAb signatures could be utilized to inform HIV-1 Env immunogens design to elicit antibody responses with greater neutralization breadth.

## Application of bNAbs: An Arsenal of Protective Potential

### Passive Immunization

As an alternative to vaccine development, passive immunization is being explored as a potential therapeutic for treatment and prevention of HIV-1 infection. In early preclinical animal studies involving passive transfusion of HIV-1 neutralizing sera and first generation bNAbs, relatively effective protection was conferred against certain HIV-1 strains (120). However, due to the poor breadth and potency of first generation bNAbs, and primarily the cost of manufacture and feasibility, passive immunization was not pursued further (121). Nevertheless, with the discovery of more potent and broad second generation bNAbs, such as VRC01 and its clonal variants, research into passive immunization has resurfaced and has demonstrated prevention and treatment potential against lentiviral infection in macaques, humanized mice, and humans (122–132). In one such study, intravenous transfusion of PGT121 mediated sterilizing immunity against SHIV in monkeys at lower concentrations than those observed in previous studies (132). Similarly, administered in monkeys, VRC01 was providing complete protection against high-dose SHIV vaginal and rectal challenge (133). In contrast to a high-dose challenge model, humans are typically exposed to small but multiple doses of HIV-1 *via* a mucosal route. In a repeated low-dose SHIV intrarectal challenge model, a single administration of bNAb (VRC01, 3BNC117, 10-1074, or VRC01-LS) to macaques was protective against infection for several months. The duration of protection was correlated with the antibody potency and half-life (125). Two V2-specific antibodies, PGDM1400 and CAP256-VRC26.25, have demonstrated high potency and neutralization breadth against HIV-1. Not much exploration has been pursued into V2-specific bNAbs, however PGDM1400 has been shown to be fully protective at 0.4mg/kg dose against SHIV challenge in a macaque model. Similarly, CAP256-VRC26.25 conferred complete immunity at a smaller dosage of 0.08 mg/kg, and thus showing the potential for V2-specific bNAbs as potential passive immunization therapeutic agents (134).

Apart from monkeys, two human clinical trials have also determined that VRC01 passive transfusion is a safe and effective endeavor (135, 136). Passive immunization using VRC01 is now being tested on a larger scale by HIV Vaccine Trials Network (HVTN) and HIV Prevention Trials Network (Antibody Mediated Prevention) and involves 2700 high-risk homosexual men in the Americas (NCT02716675) and 1,900 heterosexual women in Africa (NCT02568215). Participants are intravenously administered with either 10 mg/kg or 30 mg/kg of VRC01 once every week for 8 weeks. A protective titers for passive immunization will be determined by comparing number of infections in the dose groups and a protective efficacy will be determined by comparing number of infections in VRC01 groups to placebo (137). Another CD4-binding bNAb, 3BNC117, was tested in a dose escalation phase 1 clinical trial (NCT02018510) involving uninfected and HIV-1-infected individuals. A single 30mg/kg administration of 3BNC117 was shown to reduce viremia by 0.8-2.5 log_10_ and demonstrated favorable pharmacokinetics. However, emergence of resistant viral strains was evident in some cases (124). In another small-scale clinical trial (NCT02511990), 33 subjects received a single intravenous infusion of 10-1074 bNAb, which was well tolerated with a half-life of 25 days in uninfected and 12.8 days in HIV-1-infected individuals. Of the 13 HIV-1-infected patients enrolled in the study, 11 participants showed a rapid decline and subsequent control of viremia (138, 139). Currently, the pharmacokinetics and anti-viral activity of PGDM1400 and PGT121 is under investigation in HIV-1-infected and HIV-1-uninfected adults in a Phase I clinical trial (NCT03205917). Ongoing clinical trials using various anti-HIV-1 bNAbs are shown in **Table 2**.

**Table 2:** Ongoing clinical trials by using various anti-HIV-1 antibodies.

Trial registry identifier	Antibody	Sponsor	Phase	Estimated end date	Number of participants
NCT04801758	VRC01	HIV Vaccine Trials Network	N/A	Jun 2022	30
NCT04319367	10-1074-LS + 3BNC117-LS	Imperial College London	Phase II	Mar 2025	72
NCT04404049	UB-421	UBP Greater China (Shanghai) Co., Ltd	Phase II	Jun 2024	39
NCT03743376	UB-421	United BioPharma	Phase II	Dec2021	31
NCT03147859	vedolizumab	Ottawa Hospital Research Institute	Phase II	Dec2021	24
NCT03721510	PGT121 + VRC07-523LS +/- PGDM1400	International AIDS Vaccine Initiative	Phase I/IIa	Oct 2022	18
NCT03208231	VRC01	NIAID	Phase I/II	Feb 2021	68
NCT03707977	VRC01LS + 10-1074	NIAID	Phase I/II	Oct 2021	40
NCT03554408	10-1074-LS + 3BNC117-LS	Rockefeller University	Phase I	Jun 2021	75
NCT03571204	3BNC117 + 10-1074	NIAID	Phase I	Jun 2021	27
NCT03526848	3BNC117 + 10-1074	Rockefeller University	Phase I	Apr 2022	26
NCT04250636	3BNC117-LS + 10-1074-LS	Rockefeller University	Phase I	Feb 2022	10
NCT03374202	AAV8-VRC07	NIAID	Phase I	Mar 2027	25
NCT03705169	SAR441236	NIAID	Phase I	Feb 2022	84
NCT02591420	VRC01	NIAID	Phase I	Mar 2021	24

N/A, not applicable.

Further studies are required to determine optimal treatment regimens for passive immunization. These investigations can differ based on pharmacodynamics and pharmacokinetics in serum, as well as the bNAb HIV-1-specific pharmacodynamic properties, such as neutralization efficacy, susceptibility to viral resistance, and its capacity to mediate viral and antigen trafficking, processing, and presentation. Regimens can also depend on the patient’s viral load, variants present at the time of therapeutic administration, as well as their sensitivity to specific bNAbs (139). Collectively, these factors will provide a more personalized regimen that will ensure viral load suppression, with reduced susceptibility for viral evasion, as long as adherence is maintained.

Although passive immunization shows promise, many factors contribute to raise feasibility concerns for its application as a large-scale human prophylactic and therapeutic. First, passive immunization involves high doses of antibodies over a long period of time. Consequently, it requires relatively large amounts of expensive reagents, inflating the cost to treat one patient. Additionally, monoclonal antibodies are difficult to produce in large amounts. Therefore, passive immunization is currently limited by the absence of a cost-effective and high yield monoclonal antibody producing process (140). Lastly, immunity conferred by passive immunization is not long-lived and would require frequent and regular re-administration depending on the relatively short half-life of antibodies (141). This can lead to a potential adherence problem as seen with currently employed ART treatment. Taking a “therapeutic holiday” can theoretically allow escape variants to develop, making subsequent treatment with the same antibody difficult.

### Gene Transfer Therapy

To overcome barriers associated with passive immunization, using gene transfer technology to provide hosts with an anti-HIV-1 bNAb gene is now becoming a very attractive strategy. Previously successful gene replacement therapies have all used viral vectors for gene delivery, since viruses are highly adapted for gene delivery to their host cells. These have either involved direct viral vector injection to target tissues, or modification of cells in culture by viral vectors, followed by cell expansion and injection. After injection, the antibody gene in the transduced target cell can direct endogenous expression of the antibody molecule, and serve as a depot to synthesize bNAbs that are distributed to the circulatory system. The host is now armed with a potent bNAb against HIV-1 that bypasses the adaptive immune system (140).

A promising novel gene replacement methodology, vectored immunoprophylaxis (VIP), is capable of *in vivo* bNAb gene delivery by a single injection of adeno-associated virus (AAV) vector. To evaluate and validate its plausibility, several animal model studies have been conducted. Interestingly, an injection of immunodeficient mice with AAV8 vector, encoding a full length bNAb gene for either b12, 2G12, 4E10, 2F5, or VRC01, was demonstrated to achieve peak antibody production in serum by 6 weeks, followed by a decrease that was maintained at a consistent level for the remainder of the study. The immunodeficient mice were then injected with human-derived PBMC for 2 weeks and were subsequently challenged with 10ng of NL4-3 HIV-1 strain intravenously. A VRC01 titer of 8.3μg/ml was able to be achieved and provided complete protection against the HIV-1 challenge, thus demonstrating a proof of concept of VIP (142).

However, the intravenous HIV-1 injection model is not entirely representative to human infection, which establishes through the mucosal route. Therefore, to model HIV-1 human infection, a bone marrow-liver-thymic (BLT) chimeric mouse model was intravaginally challenged with a low dose of HIV-1 JR-CSF strain. BLT mice that were given VIP to express VRC01 were highly resistant to this challenge, elucidating the successful VIP-mediated delivery of bNAb and its efficacy of protection against HIV-1 (143). Efficacy evaluation in the nonhuman primate (NHP) model has also been carried out by SIV challenge of macaque monkeys, in which VIP-mediated bNAb expression was detected for over 6 years, providing protection against infection (144). A recent study with the NHP model demonstrated that, after receiving the AAV-encoded multiple bNAbs (3BNC117, 10-1074, and 10E8), two antibodies (3BNC117 and 10-1074) maintained high level (50-150 mug/mL) in one out of four SHIV-AD8 infected monkeys over two years, and resulted in undetectable plasma viremia over three years (145).

Encouraging results in animal models has rallied substantial interest to push VIP gene transfer therapy to clinical trials. Priddy et al. reported the first-in-human phase 1 trial (NCT01937455) with rAAV1-PG9DP (encoding PG9) in 21 volunteers performed in the UK (146). There were only mild or moderate reactions without intervention in the participants. PG9 was detected by RT-PCR in muscle biopsy samples in four volunteers and showed HIV-1 neutralization activity in the serum samples. However, ELISA detected no PG9 in serum. On the other hand, ten volunteers in the higher dose groups were detected PG9 anti-drug antibody (ADA), anti-AAV1 antibodies and AAV1-specific T-cell responses. As previously mentioned, anti-HIV-1 bNAbs undergo high levels of somatic hypermutation. Such highly mutated regions serve as potential targets for anti-idiotypic responses, which in turn would diminish their protective activity and lead to the eventual loss of transgene expression (144). Additionally, some bNAbs have a polyreactive characteristic, which allows them to bind to human antigens with high affinity as well (147, 148). For example, VRC01, VRC02, CH106, and CH103 can bind to human ubiquitin ligase E3A with high affinity, with avidity correlating to neutralization breadth of the antibody (149). The combination of auto-reactive bNAbs with the long-term antibody expression achieved by gene therapy creates a potential for autoimmunity. Therefore, before application of VIP gene transfer therapy into clinical use, challenges associated with gene transfer therapy must be addressed, i.e. how to achieve a protective bNAb level in transmission sites while minimizing the side effects caused by long-term bNAb expression.

## Challenges of bNAb Application and Possible Solutions

### Half-Life of bNAbs

Improving the half-life of bNAbs may improve the ability to prevent infection by neutralization due to its longer lasting presence. Additionally, viral rebound occurs rapidly upon bNAbs’ decay (139), which presents opportunities for viral evolution. For passive immunization applications, improving the half-life may reduce adherence complications, in turn reducing the possibility for viral evolution, and preventing both viremia and viral resistance. Previously, bNAb levels have been shown to decay more rapidly in HIV-1-infected individuals compared to the uninfected, which may be attributed to the formation of antibody-virus complexes that are readily recognized and cleared (139). Taking this into account, serum half-life needs to be improved to maintain concentrations required for protective potential and constrain viral evolution in those who are already HIV-1-infected. One such methodology, involving Fc engineering, introduces two mutations encoding amino acid substitutions (M428L and N434S, collectively known as LS) into genes encoding Fc domains of VRC01. Compared to its wild-type counterpart, VRC01-LS (now being explored in Phase I clinical trials: NCT02840474, NCT02256631; has been completed in Phase I clinical trials: NCT02797171, NCT02599896) demonstrated a 3-fold increase in its half-life, accompanied by an increase in its ability to translocate to mucosal tissues and protection against high-dose rectal challenge in non-human primates (150–152). Similarly, 3BNC117-LC and 10-1074-LC have also demonstrated to have a 2.0 and 3.8-fold increase in half life, respectively (125).

### HIV-1 Resistance to bNAbs

HIV-1 resistance to bNAb neutralization has been ever increasing over the course of the epidemic. In a previous study, HIV-1 variants isolated from recently infected individuals and from those infected early in the epidemic were tested for their sensitivity to first and second generation bNAbs. It was demonstrated that HIV-1 variants from recently infected individuals showed a decrease in sensitivity to neutralization to b12, but not to 2G12, 2F5, or 4E10. Of the 21, at least one variant from recently infected patients also showed decreased neutralization sensitivity to PG16, PG9, and VRC01, one of the most potent bNAbs thus far described (153). It has been previously established that neutralization escape from single antibody administration can occur rapidly (132). Nonetheless, a cocktail of 3-4 bNAbs can be used to reduce chances of resistance development while being capable of neutralizing 100% of viruses. Although bNAbs can prevent and control an HIV-1 infection, their breadth is invariably too limited for use as monotherapy. To address this problem, bispecific and trispecific antibodies have been developed. A recent study reported that a new class of bispecific antibodies targeting the V2-glycan (apex) and V3-glycan regions of the HIV-1 Env showed more potent neutralization than their parental bNAbs (154). Despite its positive implications, the use of more than one bNAb in therapeutic and phrophylactic strategies increases the cost of the product, raising feasibility concerns.

### Anti-Drug Antibodies

Due to its longer half-life and its ability to efficiently mediate antibody effector functions, the IgG1 subtype is usually selected for AAV delivery of bNAbs. However, it has been discovered that IgG1-Fc is responsible for the generation of anti-drug antibodies (ADAs) which results in loss of antibody expression. To circumvent this issue, Gardner et al. (155) utilized a rhesus IgG2-Fc domain to generate an antibody-like molecule eCD4-Ig to express four anti-HIV-1 bNAbs: NIH45-46, 3BNC117, PGT121 and 10-1074. To investigate the ADA and efficacy of these antibodies with either IgG1-Fc or IgG2-Fc domain, they compared AAV1 expression of these antibodies in macaques, and found that the macaques expressing IgG2-isotyped bNAbs were protected from two SHIV-AD8 challenges, but not the macaques expressing IgG1, and observed significantly lower ADA in the former macaques. These data suggest that, when using AAV1 as an expression vector, IgG2-isotyped bNAbs are less immunogenic than their IgG1 counterparts,

### Autoimmune Diseases

As previously mentioned, many bNAbs possess autoreactive potential, which has been demonstrated using autoimmune diagnosing assays and testing on arrays of human proteins. In studies involving the tracking of maturation from initial B cell arrangement to breadth development, a correlation was observed between autoreactivity and neutralization breadth. In addition, CH98, a CD4bs targeting bNAb, was isolated from a person living with an HIV-1 infection and systemic lupus erythematosus (SLE). When testing the autoreactivity of CH98, it was found to be capable of binding dsDNA, suggesting that some bNAbs derive from an autoreactive pool of B cells (156). Autoreactivity may pose a problem in gene transfer therapy, where an individual is endowed the ability to endogenously and constitutively produce these bNAbs. A strategy for controlling the endogenous bNAb expression may alleviate some degree of autoimmunity concerns.

### Cost-Effectiveness of bNAbs for Protection

The combination of the short half-life (thus requiring repeated administrations of bNAbs in passive immunization) and cost of the clinical-grade reagents required to manufacture bNAbs raisefeasibility concerns and impede its applications on a population-wide scale. Targeting bNAbs to anatomical sites of exposure may reduce the number and volume of doses required to provide protection against HIV-1 infection, consequently reducing the cost. Up to now, it remains uncertain whether either infection or vaccination with HIV-1 Env can generate protective mucosal or systemic IgA responses. However, it is crucial to induce long-lived effective mucosal antibody responses for providing protection in mucosal layer. In recent years, important progress has been made in unveiling events that occur during the exposure of HIV-1 to the mucosal surface, one of the predominant infection routes. It is unclear how the initial infection is established in the mucosal layer or mucosally. However, it has been demonstrated that HIV-1 can infect the vaginal, ectocervical, endocervical, and endometrial mucosa. The vagina and ectocervix are covered by an intact multilayered squamous epithelium, providing an intrinsic mechanical protection, while the endometrium and endocervix are covered by a single layer of columnar epithelium. Regardless, HIV-1 can penetrate corresponding cells and cause infection in all four regions mentioned above. Therefore, in addition to potentially reducing the cost of bNAb-involved treatment, targeting bNAbs to the mucosa will increase protection by serving as an alternative line of defense to mechanical barriers (157, 158).

Antibody engineering strategies have been explored to improve and establish bNAb use for mucosal immunity. One strategy involves engineering the Fc region such that it enhances binding to FcRn (neonatal FcR that is involved in IgG transepithelial transport) and pIgR (polymeric immunoglobulin receptor that is involved in IgA transepithelial transport). Anti-HIV-1 bNAb variants modified to improve binding ability have been demonstrated to have an extended half-life, enhanced localization and persistence at mucosal surfaces, and superior protection from intrarectal SHIV challenge in macaques (151). Designing IgA and chimeric IgGA variant bNAbs may also increase localization and protection in mucosa. Engineering gene transfer therapy to produce a localized response in mucosal tissue may also be beneficial. Additionally, combination therapy of cART and bNAbs may be a superior substitution to maintain viral suppression in HIV-1-infected humans.

### Potential Suboptimal Efficacy in Virus Cell-to-Cell Transmission

Studies have reported that HIV-1 infects target cells *via* two mechanisms: cell-free virus spread or cell-to-cell transmission (159, 160). More importantly, HIV-1 cell-to-cell transmission showed higher transmission efficiency than cell-free virus spread (161). A number of studies have confirmed that bNAbs could efficiently inhibit intravenous and mucosal infection caused by cell-free HIV-1 or SHIV challenge (125, 162, 163). However, previous studies have demonstrated that cell-mediated HIV-1 transmission is less sensitive to antiretroviral drugs and bNAbs than cell-free viral infection (164, 165). The activities of bNAb-mediated inhibition of cell-to-cell transmission are likely associated with the steric-hinerance effect caused by virological synapse (166). Thus, antibodies with smaller size may show more potent neutralization activity in HIV-1 cell-to-cell dissemination. Duncan et al. found that 10E8 Fab fragment presented more efficient neutralization activity than the original 10E8 in HIV-1 cell-to-cell transmission (167). Additionally, some studies indicated that the activity of bNAbs-mediated inhibition in cell-to-cell transmission mainly depended on mode of action and virus strains (168, 169). Overall, further understanding of the mechanisms of HIV-1 cell-to-cell transmission may promote the future application of bNAbs for inhibiting HIV-1 spread.

## Conclusion

The progress into characterizing the role and applicability of bNAbs in HIV-1 treatment over the years has rapidly accumulated. More focus and investigation into understanding the mechanism of bNAb generation, however, may be beneficial on several fronts, including the development of an effective vaccine that can elicit a potent bNAb response. Passive immunization over the years has also been improved, and its transition into a population-wide therapeutic can potentially be eased with reduced production cost. Additionally, research in cancer immunology has led to the establishment of gene transfer therapy, which has become a promising approach for HIV-1 treatment. Efficacy of both passive immunization and gene transfer therapy may significantly benefit from the discovery of more potent bNAbs. Moreover, bNAb-induced protection may be less effective in cell-cell transmission, and therefore, increasing our knowledge on mucosal transmission events and HIV-1 spread through infected cells will provide a strong foundation for improvement.

## Data Availability Statement

The raw data supporting the conclusions of this article will be made available by the authors, without undue reservation.

## Author Contributions 

CD, DP, YM, JW, and YG wrote sections and JM and YG reviewed the article. All authors contributed to the article and approved the submitted version.

## Funding

This work was funded by awards from Anhui Science and Technology Bureau (201904b11020044), CIHR (HBF143165, PJT 149075) and Fundamental Research Funds for the Central Universities (WK9110000167).

## Conflict of Interest

The authors declare that the research was conducted in the absence of any commercial or financial relationships that could be construed as a potential conflict of interest.

## References

[1] Sierra S Kupfer B Kaiser R. Basics of the Virology of HIV-1 and its Replication. J Clin Virol (2005) 34(4):233–44. 10.1016/j.jcv.2005.09.004 16198625

[2] Luckheeram RV Zhou R Verma AD Xia B. Cd4(+)T Cells: Differentiation and Functions. Clin Dev Immunol (2012) 2012:925135. 10.1155/2012/925135 22474485 PMC3312336

[3] McMichael AJ Rowland-Jones SL. Cellular Immune Responses to HIV. Nature (2001) 410(6831):980–7. 10.1038/35073658 11309628

[4] Kasper LH Buzoni-Gatel D. Some Opportunistic Parasitic Infections in AIDS: Candidiasis, Pneumocystosis, Cryptosporidiosis, Toxoplasmosis. Parasitol Today (1998) 14(4):150–6. 10.1016/S0169-4758(97)01212-X 17040733

[5] Centers for Disease C. Pneumocystis Pneumonia–Los Angeles. MMWR Morb Mortal Wkly Rep (1981) 30(21):250–2. 6265753

[6] Piot P Quinn TC. Response to the AIDS Pandemic–a Global Health Model. N Engl J Med (2013) 368(23):2210–8. 10.1056/NEJMra1201533 PMC3777557 23738546

[7] Quinn TC. HIV Epidemiology and the Effects of Antiviral Therapy on Long-Term Consequences. AIDS (2008) 22 Suppl 3:S7–12. 10.1097/01.aids.0000327510.68503.e8 PMC2753265 18845925

[8] Andrabi R Voss JE Liang CH Briney B McCoy LE Wu CY. Identification of Common Features in Prototype Broadly Neutralizing Antibodies to HIV Envelope V2 Apex to Facilitate Vaccine Design. Immunity (2015) 43(5):959–73. 10.1016/j.immuni.2015.10.014 PMC4654981 26588781

[9] Arien KK Vanham G Arts EJ. Is HIV-1 Evolving to a Less Virulent Form in Humans? Nat Rev Microbiol (2007) 5(2):141–51. 10.1038/nrmicro1594 PMC7097722 17203103

[10] Autran B Carcelain G Li TS Blanc C Mathez D Tubiana R. Positive Effects of Combined Antiretroviral Therapy on CD4+ T Cell Homeostasis and Function in Advanced HIV Disease. Science (1997) 277(5322):112–6. 10.1126/science.277.5322.112 9204894

[11] Komanduri KV Viswanathan MN Wieder ED Schmidt DK Bredt BM Jacobson MA. Restoration of Cytomegalovirus-Specific CD4+ T-Lymphocyte Responses After Ganciclovir and Highly Active Antiretroviral Therapy in Individuals Infected With HIV-1. Nat Med (1998) 4(8):953–6. 10.1038/nm0898-953 9701250

[12] Lederman MM Connick E Landay A Kuritzkes DR Spritzler J St Clair M. Immunologic Responses Associated With 12 Weeks of Combination Antiretroviral Therapy Consisting of Zidovudine, Lamivudine, and Ritonavir: Results of AIDS Clinical Trials Group Protocol 315. J Infect Dis (1998) 178(1):70–9. 10.1086/515591 9652425

[13] Broder S Hoffman SL Hotez PJ. Cures for the Third World’s Problems: The Application of Genomics to the Diseases Plaguing the Developing World may Have Huge Medical and Economic Benefits for Those Countries and Might Even Prevent Armed Conflict. EMBO Rep (2002) 3(9):806–12. 10.1093/embo-reports/kvf187 PMC1084240 12223456

[14] Hallenberger S Bosch V Angliker H Shaw E Klenk HD Garten W. Inhibition of Furin-Mediated Cleavage Activation of HIV-1 Glycoprotein gp160. Nature (1992) 360(6402):358–61. 10.1038/360358a0 1360148

[15] Wyatt R Sodroski J. The HIV-1 Envelope Glycoproteins: Fusogens, Antigens, and Immunogens. Science (1998) 280(5371):1884–8. 10.1126/science.280.5371.1884 9632381

[16] Chan DC Kim PS. HIV Entry and its Inhibition. Cell (1998) 93(5):681–4. 10.1016/S0092-8674(00)81430-0 9630213

[17] Wilen CB Tilton JC Doms RW. HIV: Cell Binding and Entry. Cold Spring Harb Perspect Med (2012) 2(8):a006866. 10.1101/cshperspect.a006866 22908191 PMC3405824

[18] Wei X Decker JM Wang S Hui H Kappes JC Wu X. Antibody Neutralization and Escape by HIV-1. Nature (2003) 422(6929):307–12. 10.1038/nature01470 12646921

[19] Richman DD Wrin T Little SJ Petropoulos CJ. Rapid Evolution of the Neutralizing Antibody Response to HIV Type 1 Infection. Proc Natl Acad Sci USA (2003) 100(7):4144–9. 10.1073/pnas.0630530100 PMC153062 12644702

[20] Shcherbakov DN Bakulina AY Karpenko LI Ilyichev AA. Broadly Neutralizing Antibodies Against HIV-1 As a Novel Aspect of the Immune Response. Acta Naturae (2015) 7(4):11–21. 10.32607/20758251-2015-7-4-11-21 26798488 PMC4717246

[21] Korber B Gaschen B Yusim K Thakallapally R Kesmir C Detours V. Evolutionary and Immunological Implications of Contemporary HIV-1 Variation. Br Med Bull (2001) 58:19–42. 10.1093/bmb/58.1.19 11714622

[22] Walker BD Burton DR. Toward an AIDS Vaccine. Science (2008) 320(5877):760–4. 10.1126/science.1152622 18467582

[23] Peeters M Sharp PM. Genetic Diversity of HIV-1: The Moving Target. AIDS (2000) 14 Suppl 3:S129–140. 11086856

[24] Lihana RW Ssemwanga D Abimiku A Ndembi N. Update on HIV-1 Diversity in Africa: A Decade in Review. AIDS Rev (2012) 14(2):83–100. 22627605

[25] Kimura T Wang XH Williams C Zolla-Pazner S Gorny MK. Human Monoclonal Antibody 2909 Binds to Pseudovirions Expressing Trimers But Not Monomeric HIV-1 Envelope Proteins. Hum Antibodies (2009) 18(1-2):35–40. 10.3233/HAB-2009-0200 19478397 PMC3887469

[26] Kwong PD Doyle ML Casper DJ Cicala C Leavitt SA Majeed S. HIV-1 Evades Antibody-Mediated Neutralization Through Conformational Masking of Receptor-Binding Sites. Nature (2002) 420(6916):678–82. 10.1038/nature01188 12478295

[27] Wyatt R Kwong PD Desjardins E Sweet RW Robinson J Hendrickson WA. The Antigenic Structure of the HIV Gp120 Envelope Glycoprotein. Nature (1998) 393(6686):705–11. 10.1038/31405 9641684

[28] Scanlan CN Offer J Zitzmann N Dwek RA. Exploiting the Defensive Sugars of HIV-1 for Drug and Vaccine Design. Nature (2007) 446(7139):1038–45. 10.1038/nature05818 17460665

[29] Falkowska E Le KM Ramos A Doores KJ Lee JH Blattner C. Broadly Neutralizing HIV Antibodies Define a Glycan-Dependent Epitope on the Prefusion Conformation of gp41 on Cleaved Envelope Trimers. Immunity (2014) 40(5):657–68. 10.1016/j.immuni.2014.04.009 PMC4070425 24768347

[30] McCaffrey RA Saunders C Hensel M Stamatatos L. N-Linked Glycosylation of the V3 Loop and the Immunologically Silent Face of gp120 Protects Human Immunodeficiency Virus Type 1 SF162 From Neutralization by Anti-gp120 and Anti-gp41 Antibodies. J Virol (2004) 78(7):3279–95. 10.1128/jvi.78.7.3279-3295.2004 PMC371088 15016849

[31] Wang W Nie J Prochnow C Truong C Jia Z Wang S. A Systematic Study of the N-Glycosylation Sites of HIV-1 Envelope Protein on Infectivity and Antibody-Mediated Neutralization. Retrovirology (2013) 10:14. 10.1186/1742-4690-10-14 23384254 PMC3648360

[32] Quinones-Kochs MI Buonocore L Rose JK. Role of N-Linked Glycans in a Human Immunodeficiency Virus Envelope Glycoprotein: Effects on Protein Function and the Neutralizing Antibody Response. J Virol (2002) 76(9):4199–211. 10.1128/jvi.76.9.4199-4211.2002 PMC155056 11932385

[33] Huang X Jin W Hu K Luo S Du T Griffin GE. Highly Conserved HIV-1 gp120 Glycans Proximal to CD4-Binding Region Affect Viral Infectivity and Neutralizing Antibody Induction. Virology (2012) 423(1):97–106. 10.1016/j.virol.2011.11.023 22192629

[34] Kolchinsky P Kiprilov E Sodroski J. Increased Neutralization Sensitivity of CD4-Independent Human Immunodeficiency Virus Variants. J Virol (2001) 75(5):2041–50. 10.1128/JVI.75.5.2041-2050.2001 PMC114788 11160708

[35] Townsley S Li Y Kozyrev Y Cleveland B Hu SL. Conserved Role of an N-Linked Glycan on the Surface Antigen of Human Immunodeficiency Virus Type 1 Modulating Virus Sensitivity to Broadly Neutralizing Antibodies Against the Receptor and Coreceptor Binding Sites. J Virol (2016) 90(2):829–41. 10.1128/JVI.02321-15 PMC4702696 26512079

[36] Dhillon AK Donners H Pantophlet R Johnson WE Decker JM Shaw GM. Dissecting the Neutralizing Antibody Specificities of Broadly Neutralizing Sera From Human Immunodeficiency Virus Type 1-Infected Donors. J Virol (2007) 81(12):6548–62. 10.1128/JVI.02749-06 PMC1900098 17409160

[37] Doria-Rose NA Klein RM Manion MM O’Dell S Phogat A Chakrabarti B. Frequency and Phenotype of Human Immunodeficiency Virus Envelope-Specific B Cells From Patients With Broadly Cross-Neutralizing Antibodies. J Virol (2009) 83(1):188–99. 10.1128/JVI.01583-08 PMC2612342 18922865

[38] Stephenson KE Barouch DH. Broadly Neutralizing Antibodies for HIV Eradication. Curr HIV/AIDS Rep (2016) 13(1):31–7. 10.1007/s11904-016-0299-7 PMC4779134 26841901

[39] Havenar-Daughton C Lee JH Crotty S. Tfh Cells and HIV bnABs, an Immunodominance Model of the HIV Neutralizing Antibody Generation Problem. Immunol Rev (2017) 275(1):49–61. 10.1111/imr.12512 28133798

[40] Crotty S. T Follicular Helper Cell Differentiation, Function, and Roles in Disease. Immunity (2014) 41(4):529–42. 10.1016/j.immuni.2014.10.004 PMC4223692 25367570

[41] Burton DR Mascola JR. Antibody Responses to Envelope Glycoproteins in HIV-1 Infection. Nat Immunol (2015) 16(6):571–6. 10.1038/ni.3158 PMC4834917 25988889

[42] Rajasekaran N Chester C Yonezawa A Zhao X Kohrt HE. Enhancement of Antibody-Dependent Cell Mediated Cytotoxicity: A New Era in Cancer Treatment. Immunotargets Ther (2015) 4:91–100. 10.2147/ITT.S61292 27471715 PMC4918249

[43] Frost SD Wrin T Smith DM Kosakovsky Pond SL Liu Y Paxinos E. Neutralizing Antibody Responses Drive the Evolution of Human Immunodeficiency Virus Type 1 Envelope During Recent HIV Infection. Proc Natl Acad Sci USA (2005) 102(51):18514–9. 10.1073/pnas.0504658102 PMC1310509 16339909

[44] Murphy MK Yue L Pan R Boliar S Sethi A Tian J. Viral Escape From Neutralizing Antibodies in Early Subtype A HIV-1 Infection Drives an Increase in Autologous Neutralization Breadth. PloS Pathog (2013) 9(2):e1003173. 10.1371/journal.ppat.1003173 23468623 PMC3585129

[45] Fuchs SP Desrosiers RC. Promise and Problems Associated With the Use of Recombinant AAV for the Delivery of Anti-HIV Antibodies. Mol Ther Methods Clin Dev (2016) 3:16068. 10.1038/mtm.2016.68 28197421 PMC5289440

[46] Zhou T Georgiev I Wu X Yang ZY Dai K Finzi A. Structural Basis for Broad and Potent Neutralization of HIV-1 by Antibody VRC01. Science (2010) 329(5993):811–7. 10.1126/science.1192819 PMC2981354 20616231

[47] Diskin R Scheid JF Marcovecchio PM West AP Jr. Klein F Gao H. Increasing the Potency and Breadth of an HIV Antibody by Using Structure-Based Rational Design. Science (2011) 334(6060):1289–93. 10.1126/science.1213782 PMC3232316 22033520

[48] McLellan JS Pancera M Carrico C Gorman J Julien JP Khayat R. Structure of HIV-1 gp120 V1/V2 Domain With Broadly Neutralizing Antibody PG9. Nature (2011) 480(7377):336–43. 10.1038/nature10696 PMC3406929 22113616

[49] Mouquet H Scharf L Euler Z Liu Y Eden C Scheid JF. Complex-Type N-Glycan Recognition by Potent Broadly Neutralizing HIV Antibodies. Proc Natl Acad Sci USA (2012) 109(47):E3268–77. 10.1073/pnas.1217207109 PMC3511153 23115339

[50] Walker LM Phogat SK Chan-Hui PY Wagner D Phung P Goss JL. Broad and Potent Neutralizing Antibodies From an African Donor Reveal a New HIV-1 Vaccine Target. Science (2009) 326(5950):285–9. 10.1126/science.1178746 PMC3335270 19729618

[51] Walker LM Huber M Doores KJ Falkowska E Pejchal R Julien JP. Broad Neutralization Coverage of HIV by Multiple Highly Potent Antibodies. Nature (2011) 477(7365):466–70. 10.1038/nature10373 PMC3393110 21849977

[52] Huang J Ofek G Laub L Louder MK Doria-Rose NA Longo NS. Broad and Potent Neutralization of HIV-1 by a gp41-Specific Human Antibody. Nature (2012) 491(7424):406–12. 10.1038/nature11544 PMC4854285 23151583

[53] Scharf L Scheid JF Lee JH West AP Jr. Chen C Gao H. Antibody 8anc195 Reveals a Site of Broad Vulnerability on the HIV-1 Envelope Spike. Cell Rep (2014) 7(3):785–95. 10.1016/j.celrep.2014.04.001 PMC4109818 24767986

[54] Huang J Kang BH Pancera M Lee JH Tong T Feng Y. Broad and Potent HIV-1 Neutralization by a Human Antibody That Binds the gp41-gp120 Interface. Nature (2014) 515(7525):138–+. 10.1038/nature13601 PMC4224615 25186731

[55] Schoofs T Barnes CO Suh-Toma N Golijanin J Schommers P Gruell H. Broad and Potent Neutralizing Antibodies Recognize the Silent Face of the HIV Envelope. Immunity (2019) 50(6):1513–+. 10.1016/j.immuni.2019.04.014 PMC6591006 31126879

[56] Zhou T Zheng A Baxa U Chuang G-Y Georgiev IS Kong R. A Neutralizing Antibody Recognizing Primarily N-Linked Glycan Targets the Silent Face of the HIV Envelope. Immunity (2018) 48(3):500–+. 10.1016/j.immuni.2018.02.013 PMC6421865 29548671

[57] Kong R Xu K Zhou TQ Acharya P Lemmin T Liu K. Fusion Peptide of HIV-1 as a Site of Vulnerability to Neutralizing Antibody. Science (2016) 352(6287):828–33. 10.1126/science.aae0474 PMC4917739 27174988

[58] Binley JM Lybarger EA Crooks ET Seaman MS Gray E Davis KL. Profiling the Specificity of Neutralizing Antibodies in a Large Panel of Plasmas From Patients Chronically Infected With Human Immunodeficiency Virus Type 1 Subtypes B and C. J Virol (2008) 82(23):11651–68. 10.1128/JVI.01762-08 PMC2583680 18815292

[59] Gray ES Moore PL Choge IA Decker JM Bibollet-Ruche F Li H. Neutralizing Antibody Responses in Acute Human Immunodeficiency Virus Type 1 Subtype C Infection. J Virol (2007) 81(12):6187–96. 10.1128/JVI.00239-07 PMC1900112 17409164

[60] Simek MD Rida W Priddy FH Pung P Carrow E Laufer DS. Human Immunodeficiency Virus Type 1 Elite Neutralizers: Individuals With Broad and Potent Neutralizing Activity Identified by Using a High-Throughput Neutralization Assay Together With an Analytical Selection Algorithm. J Virol (2009) 83(14):7337–48. 10.1128/JVI.00110-09 PMC2704778 19439467

[61] Wu X Yang ZY Li Y Hogerkorp CM Schief WR Seaman MS. Rational Design of Envelope Identifies Broadly Neutralizing Human Monoclonal Antibodies to HIV-1. Science (2010) 329(5993):856–61. 10.1126/science.1187659 PMC2965066 20616233

[62] Corti D Langedijk JPM Hinz A Seaman MS Vanzetta F Fernandez-Rodriguez BM. Analysis of Memory B Cell Responses and Isolation of Novel Monoclonal Antibodies With Neutralizing Breadth From HIV-1-Infected Individuals. PloS One (2010) 5(1):e8805. 10.1371/journal.pone.0008805 20098712 PMC2808385

[63] Saphire EO Parren P Barbas CF Burton DR Wilson IA. Crystallization and Preliminary Structure Determination of an Intact Human Immunoglobulin, B12: An Antibody That Broadly Neutralizes Primary Isolates of HIV-1. Acta Crystallograph Section D-Biol Crystallogr (2001) 57:168–71. 10.1107/S0907444900017376 11134947

[64] Liao H-X Lynch R Zhou T Gao F Alam SM Boyd SD. Co-Evolution of a Broadly Neutralizing HIV-1 Antibody and Founder Virus. Nature (2013) 496(7446):469–+. 10.1038/nature12053 PMC3637846 23552890

[65] Huang J Kang BH Ishida E Zhou T Griesman T Sheng Z. Identification of a CD4-Binding-Site Antibody to HIV That Evolved Near-Pan Neutralization Breadth. Immunity (2016) 45(5):1108–21. 10.1016/j.immuni.2016.10.027 PMC5770152 27851912

[66] Gorman J McLellan J Yang Y Zhou T Zhu J Bangaru S. Recombinant Env Proteins That Bind the Quaternary-Specific, V1/V2-Directed PGT Antibodies. Retrovirology (2012) 9:84. 10.1186/1742-4690-9-S2-P84 23046603

[67] Kunert R Ruker F Katinger H. Molecular Characterization of Five Neutralizing Anti-HIV Type 1 Antibodies: Identification of Nonconventional D Segments in the Human Monoclonal Antibodies 2G12 and 2F5. AIDS Res Hum Retroviruses (1998) 14(13):1115–28. 10.1089/aid.1998.14.1115 9737583

[68] Cao J Bergeron L Helseth E Thali M Repke H Sodroski J. Effects Of Amino-Acid Changes In The Extracellular Domain Of The Human-Immunodeficiency-Virus Type-1 gp41 Envelope Glycoprotein. J Virol (1993) 67(5):2747–55. 10.1128/jvi.67.5.2747-2755.1993 PMC237598 8474172

[69] Chen YH Dierich MP. Identification of a Second Site in HIV-1 gp41 Mediating Binding to Cells. Immunol Lett (1996) 52(2-3):153–6. 10.1016/0165-2478(96)02603-X 8905411

[70] van Gils MJ van den Kerkhof TLGM Ozorowski G Cottrell CA Sok D Pauthner M. An HIV-1 Antibody From an Elite Neutralizer Implicates the Fusion Peptide as a Site of Vulnerability. Nat Microbiol (2017) 2(2):16199. 10.1038/nmicrobiol.2016.199 PMC5372380 27841852

[71] Ahmed Y Tian M Gao Y. Development of an Anti-HIV Vaccine Eliciting Broadly Neutralizing Antibodies. AIDS Res Ther (2017) 14(1):50. 10.1186/s12981-017-0178-3 28893278 PMC5594608

[72] Mouquet H Scheid JF Zoller MJ Krogsgaard M Ott RG Shukair S. Polyreactivity Increases the Apparent Affinity of Anti-HIV Antibodies by Heteroligation. Nature (2010) 467(7315):591–5. 10.1038/nature09385 PMC3699875 20882016

[73] Julg B Pegu A Abbink P Liu J Brinkman A Molloy K. Virological Control by the CD4-Binding Site Antibody N6 in Simian-Human Immunodeficiency Virus-Infected Rhesus Monkeys. J Virol (2017) 91(16):e00498–17. 10.1128/JVI.00498-17 PMC5533891 28539448

[74] Scheid JF Mouquet H Ueberheide B Diskin R Klein F Oliveira TY. Sequence and Structural Convergence of Broad and Potent HIV Antibodies That Mimic CD4 Binding. Science (2011) 333(6049):1633–7. 10.1126/science.1207227 PMC3351836 21764753

[75] Scheid JF Horwitz JA Bar-On Y Kreider EF Lu CL Lorenzi JC. HIV-1 Antibody 3BNC117 Suppresses Viral Rebound in Humans During Treatment Interruption. Nature (2016) 535(7613):556–60. 10.1038/nature18929 PMC5034582 27338952

[76] Moore PL Gray ES Sheward D Madiga M Ranchobe N Lai Z. Potent and Broad Neutralization of HIV-1 Subtype C by Plasma Antibodies Targeting a Quaternary Epitope Including Residues in the V2 Loop. J Virol (2011) 85(7):3128–41. 10.1128/JVI.02658-10 PMC3067856 21270156

[77] Sok D van Gils MJ Pauthner M Julien JP Saye-Francisco KL Hsueh J. Recombinant HIV Envelope Trimer Selects for Quaternary-Dependent Antibodies Targeting the Trimer Apex. Proc Natl Acad Sci U.S.A. (2014) 111(49):17624–9. 10.1073/pnas.1415789111 PMC4267403 25422458

[78] Julien JP Sok D Khayat R Lee JH Doores KJ Walker LM. Broadly Neutralizing Antibody PGT121 Allosterically Modulates CD4 Binding *via* Recognition of the HIV-1 Gp120 V3 Base and Multiple Surrounding Glycans. PloS Pathog (2013) 9(5):e1003342. 10.1371/journal.ppat.1003342 23658524 PMC3642082

[79] Kumar S Panda H Makhdoomi MA Mishra N Safdari HA Chawla H. An HIV-1 Broadly Neutralizing Antibody From a Clade C-Infected Pediatric Elite Neutralizer Potently Neutralizes the Contemporaneous and Autologous Evolving Viruses. J Virol (2019) 93(4):e01495–18. 10.1128/JVI.01495-18 PMC6364018 30429339

[80] Horwitz JA Bar-On Y Lu CL Fera D Lockhart AAK Lorenzi JCC. Non-Neutralizing Antibodies Alter the Course of HIV-1 Infection *In Vivo* . Cell (2017) 170(4):637–648 e610. 10.1016/j.cell.2017.06.048 28757252 PMC5554461

[81] Lewis GK Pazgier M DeVico AL. Survivors Remorse: Antibody-Mediated Protection Against HIV-1. Immunol Rev (2017) 275(1):271–84. 10.1111/imr.12510 PMC5642910 28133809

[82] Moog C Dereuddre-Bosquet N Teillaud JL Biedma ME Holl V Van Ham G. Protective Effect of Vaginal Application of Neutralizing and Nonneutralizing Inhibitory Antibodies Against Vaginal SHIV Challenge in Macaques. Mucosal Immunol (2014) 7(1):46–56. 10.1038/mi.2013.23 23591718

[83] Santra S Tomaras GD Warrier R Nicely NI Liao HX Pollara J. Human Non-Neutralizing HIV-1 Envelope Monoclonal Antibodies Limit the Number of Founder Viruses During SHIV Mucosal Infection in Rhesus Macaques. PloS Pathog (2015) 11(8):e1005042. 10.1371/journal.ppat.1005042 26237403 PMC4523205

[84] Anand SP Prevost J Baril S Richard J Medjahed H Chapleau JP. Two Families of Env Antibodies Efficiently Engage Fc-Gamma Receptors and Eliminate HIV-1-Infected Cells. J Virol (2019) 93(3):e01823–18. 10.1128/JVI.01823-18 PMC6340017 30429344

[85] Nutt SL Hodgkin PD Tarlinton DM Corcoran LM. The Generation of Antibody-Secreting Plasma Cells. Nat Rev Immunol (2015) 15(3):160–71. 10.1038/nri3795 25698678

[86] Pillai S Cariappa A Moran ST. Marginal Zone B Cells. Annu Rev Immunol (2005) 23:161–96. 10.1146/annurev.immunol.23.021704.115728 15771569

[87] Kurosaki T Kometani K Ise W. Memory B Cells. Nat Rev Immunol (2015) 15(3):149–59. 10.1038/nri3802 25677494

[88] Allman DM Ferguson SE Cancro MP. Peripheral B Cell Maturation. I. Immature Peripheral B Cells in Adults are Heat-Stable Antigenhi and Exhibit Unique Signaling Characteristics. J Immunol (1992) 149(8):2533–40. 1383316

[89] Allman DM Ferguson SE Lentz VM Cancro MP. Peripheral B Cell Maturation. II. Heat-Stable Antigen(hi) Splenic B Cells are an Immature Developmental Intermediate in the Production of Long-Lived Marrow-Derived B Cells. J Immunol (1993) 151(9):4431–44. 8409411

[90] Mouquet H. Antibody B Cell Responses in HIV-1 Infection. Trends Immunol (2014) 35(11):549–61. 10.1016/j.it.2014.08.007 25240985

[91] Victora GD Nussenzweig MC. Germinal Centers. Annu Rev Immunol (2012) 30:429–57. 10.1146/annurev-immunol-020711-075032 22224772

[92] Locci M Havenar-Daughton C Landais E Wu J Kroenke MA Arlehamn CL. Human Circulating PD-1+CXCR3-CXCR5+ Memory Tfh Cells are Highly Functional and Correlate With Broadly Neutralizing HIV Antibody Responses. Immunity (2013) 39(4):758–69. 10.1016/j.immuni.2013.08.031 PMC3996844 24035365

[93] Petrovas C Yamamoto T Gerner MY Boswell KL Wloka K Smith EC. CD4 T Follicular Helper Cell Dynamics During SIV Infection. J Clin Invest (2012) 122(9):3281–94. 10.1172/JCI63039 PMC3428091 22922258

[94] De Boer RJ Perelson AS. How Germinal Centers Evolve Broadly Neutralizing Antibodies: The Breadth of the Follicular Helper T Cell Response. J Virol (2017) 91(22):e00983–17. 10.1128/JVI.00983-17 PMC5660473 28878083

[95] Li L Liu Y Gorny MK. Association of Diverse Genotypes and Phenotypes of Immune Cells and Immunoglobulins With the Course of HIV-1 Infection. Front Immunol (2018) 9:2735. 10.3389/fimmu.2018.02735 30534128 PMC6275200

[96] Buckner CM Moir S Ho J Wang W Posada JG Kardava L. Characterization of Plasmablasts in the Blood of HIV-Infected Viremic Individuals: Evidence for Nonspecific Immune Activation. J Virol (2013) 87(10):5800–11. 10.1128/JVI.00094-13 PMC3648153 23487459

[97] Levesque MC Moody MA Hwang KK Marshall DJ Whitesides JF Amos JD. Polyclonal B Cell Differentiation and Loss of Gastrointestinal Tract Germinal Centers in the Earliest Stages of HIV-1 Infection. PloS Med (2009) 6(7):e1000107. 10.1371/journal.pmed.1000107 19582166 PMC2702159

[98] Liao HX Chen X Munshaw S Zhang R Marshall DJ Vandergrift N. Initial Antibodies Binding to HIV-1 gp41 in Acutely Infected Subjects are Polyreactive and Highly Mutated. J Exp Med (2011) 208(11):2237–49. 10.1084/jem.20110363 PMC3201211 21987658

[99] Moir S Ho J Malaspina A Wang W DiPoto AC O’Shea MA. Evidence for HIV-Associated B Cell Exhaustion in a Dysfunctional Memory B Cell Compartment in HIV-Infected Viremic Individuals. J Exp Med (2008) 205(8):1797–805. 10.1084/jem.20072683 PMC2525604 18625747

[100] Moir S Malaspina A Ogwaro KM Donoghue ET Hallahan CW Ehler LA. HIV-1 Induces Phenotypic and Functional Perturbations of B Cells in Chronically Infected Individuals. Proc Natl Acad Sci USA (2001) 98(18):10362–7. 10.1073/pnas.181347898 PMC56966 11504927

[101] Tomaras GD Yates NL Liu P Qin L Fouda GG Chavez LL. Initial B-Cell Responses to Transmitted Human Immunodeficiency Virus Type 1: Virion-Binding Immunoglobulin M (IgM) and IgG Antibodies Followed by Plasma Anti-Gp41 Antibodies With Ineffective Control of Initial Viremia. J Virol (2008) 82(24):12449–63. 10.1128/JVI.01708-08 PMC2593361 18842730

[102] Xu W Santini PA Sullivan JS He B Shan M Ball SC. HIV-1 Evades Virus-Specific IgG2 and IgA Responses by Targeting Systemic and Intestinal B Cells *via* Long-Range Intercellular Conduits. Nat Immunol (2009) 10(9):1008–17. 10.1038/ni.1753 PMC2784687 19648924

[103] Stacey AR Norris PJ Qin L Haygreen EA Taylor E Heitman J. Induction of a Striking Systemic Cytokine Cascade Prior to Peak Viremia in Acute Human Immunodeficiency Virus Type 1 Infection, in Contrast to More Modest and Delayed Responses in Acute Hepatitis B and C Virus Infections. J Virol (2009) 83(8):3719–33. 10.1128/JVI.01844-08 PMC2663284 19176632

[104] Boliar S Murphy MK Tran TC Carnathan DG Armstrong WS Silvestri G. B-Lymphocyte Dysfunction in Chronic HIV-1 Infection Does Not Prevent Cross-Clade Neutralization Breadth. J Virol (2012) 86(15):8031–40. 10.1128/JVI.00771-12 PMC3421653 22623771

[105] Mabvakure BM Scheepers C Garrett N Abdool Karim S Williamson C Morris L. Positive Selection at Key Residues in the HIV Envelope Distinguishes Broad and Strain-Specific Plasma Neutralizing Antibodies. J Virol (2019) 93(6):e01685–18. 10.1128/JVI.01685-18 PMC6401460 30567996

[106] Simonich CA Doepker L Ralph D Williams JA Dhar A Yaffe Z. Kappa Chain Maturation Helps Drive Rapid Development of an Infant HIV-1 Broadly Neutralizing Antibody Lineage. Nat Commun (2019) 10(1):2190. 10.1038/s41467-019-09481-7 31097697 PMC6522554

[107] Barnett SW Srivastava IK Ulmer JB Donnelly JJ Rappuoli R. Development of V2-Deleted Trimeric Envelope Vaccine Candidates From Human Immunodeficiency Virus Type 1 (HIV-1) Subtypes B and C. Microbes Infect (2005) 7(14):1386–91. 10.1016/j.micinf.2005.07.018 16275150

[108] Javaherian K Langlois AJ LaRosa GJ Profy AT Bolognesi DP Herlihy WC. Broadly Neutralizing Antibodies Elicited by the Hypervariable Neutralizing Determinant of HIV-1. Science (1990) 250(4987):1590–3. 10.1126/science.1703322 1703322

[109] LaRosa GJ Davide JP Weinhold K Waterbury JA Profy AT Lewis JA. Conserved Sequence and Structural Elements in the HIV-1 Principal Neutralizing Determinant. Science (1990) 249(4971):932–5. 10.1126/science.2392685 2392685

[110] Malherbe DC Doria-Rose NA Misher L Beckett T Puryear WB Schuman JT. Sequential Immunization With a Subtype B HIV-1 Envelope Quasispecies Partially Mimics the *In Vivo* Development of Neutralizing Antibodies. J Virol (2011) 85(11):5262–74. 10.1128/JVI.02419-10 PMC3094990 21430056

[111] Martin G Burke B Thai R Dey AK Combes O Ramos OH. Stabilization of HIV-1 Envelope in the CD4-Bound Conformation Through Specific Cross-Linking of a CD4 Mimetic. J Biol Chem (2011) 286(24):21706–16. 10.1074/jbc.M111.232272 PMC3122227 21487012

[112] Dosenovic P von Boehmer L Escolano A Jardine J Freund NT Gitlin AD. Immunization for HIV-1 Broadly Neutralizing Antibodies in Human Ig Knockin Mice. Cell (2015) 161(7):1505–15. 10.1016/j.cell.2015.06.003 PMC4604566 26091035

[113] Jardine JG Ota T Sok D Pauthner M Kulp DW Kalyuzhniy O. HIV-1 VACCINES. Priming a Broadly Neutralizing Antibody Response to HIV-1 Using a Germline-Targeting Immunogen. Science (2015) 349(6244):156–61. 10.1126/science.aac5894 PMC4669217 26089355

[114] Doria-Rose NA Schramm CA Gorman J Moore PL Bhiman JN DeKosky BJ. Developmental Pathway for Potent V1V2-Directed HIV-Neutralizing Antibodies. Nature (2014) 509(7498):55–62. 10.1038/nature13036 24590074 PMC4395007

[115] Haynes BF Kelsoe G Harrison SC Kepler TB. B-Cell-Lineage Immunogen Design in Vaccine Development With HIV-1 as a Case Study. Nat Biotechnol (2012) 30(5):423–33. 10.1038/nbt.2197 PMC3512202 22565972

[116] Hoot S McGuire AT Cohen KW Strong RK Hangartner L Klein F. Recombinant HIV Envelope Proteins Fail to Engage Germline Versions of Anti-CD4bs Bnabs. PloS Pathog (2013) 9(1):e1003106. 10.1371/journal.ppat.1003106 23300456 PMC3536657

[117] Jardine J Julien JP Menis S Ota T Kalyuzhniy O McGuire A. Rational HIV Immunogen Design to Target Specific Germline B Cell Receptors. Science (2013) 340(6133):711–6. 10.1126/science.1234150 PMC3689846 23539181

[118] Sok D Briney B Jardine JG Kulp DW Menis S Pauthner M. Priming HIV-1 Broadly Neutralizing Antibody Precursors in Human Ig Loci Transgenic Mice. Science (2016) 353(6307):1557–60. 10.1126/science.aah3945 PMC5404394 27608668

[119] Bricault CA Yusim K Seaman MS Yoon H Theiler J Giorgi EE. HIV-1 Neutralizing Antibody Signatures and Application to Epitope-Targeted Vaccine Design. Cell Host Microbe (2019) 26(2):296. 10.1016/j.chom.2019.07.016 31415756 PMC6706656

[120] Armbruster C Stiegler GM Vcelar BA Jager W Michael NL Vetter N. A Phase I Trial With Two Human Monoclonal Antibodies (hMAb 2F5, 2G12) Against HIV-1. Aids (2002) 16(2):227–33. 10.1097/00002030-200201250-00012 11807307

[121] Escolano A Dosenovic P Nussenzweig MC. Progress Toward Active or Passive HIV-1 Vaccination. J Exp Med (2017) 214(1):3–16. 10.1084/jem.20161765 28003309 PMC5206506

[122] Barouch DH Whitney JB Moldt B Klein F Oliveira TY Liu J. Therapeutic Efficacy of Potent Neutralizing HIV-1-Specific Monoclonal Antibodies in SHIV-Infected Rhesus Monkeys. Nature (2013) 503(7475):224–8. 10.1038/nature12744 PMC4017780 24172905

[123] Bolton DL Pegu A Wang K McGinnis K Nason M Foulds K. Human Immunodeficiency Virus Type 1 Monoclonal Antibodies Suppress Acute Simian-Human Immunodeficiency Virus Viremia and Limit Seeding of Cell-Associated Viral Reservoirs. J Virol (2016) 90(3):1321–32. 10.1128/JVI.02454-15 PMC4719604 26581981

[124] Caskey M Klein F Lorenzi JC Seaman MS West AP Jr. Buckley N. Viraemia Suppressed in HIV-1-Infected Humans by Broadly Neutralizing Antibody 3BNC117. Nature (2015) 522(7557):487–91. 10.1038/nature14411 PMC4890714 25855300

[125] Gautam R Nishimura Y Pegu A Nason MC Klein F Gazumyan A. A Single Injection of Anti-HIV-1 Antibodies Protects Against Repeated SHIV Challenges. Nature (2016) 533(7601):105–9. 10.1038/nature17677 PMC5127204 27120156

[126] Halper-Stromberg A Lu CL Klein F Horwitz JA Bournazos S Nogueira L. Broadly Neutralizing Antibodies and Viral Inducers Decrease Rebound From HIV-1 Latent Reservoirs in Humanized Mice. Cell (2014) 158(5):989–99. 10.1016/j.cell.2014.07.043 PMC4163911 25131989

[127] Hessell AJ Jaworski JP Epson E Matsuda K Pandey S Kahl C. Early Short-Term Treatment With Neutralizing Human Monoclonal Antibodies Halts SHIV Infection in Infant Macaques. Nat Med (2016) 22(4):362–8. 10.1038/nm.4063 PMC4983100 26998834

[128] Horwitz JA Halper-Stromberg A Mouquet H Gitlin AD Tretiakova A Eisenreich TR. HIV-1 Suppression and Durable Control by Combining Single Broadly Neutralizing Antibodies and Antiretroviral Drugs in Humanized Mice. Proc Natl Acad Sci U.S.A. (2013) 110(41):16538–43. 10.1073/pnas.1315295110 PMC3799352 24043801

[129] Klein F Halper-Stromberg A Horwitz JA Gruell H Scheid JF Bournazos S. HIV Therapy by a Combination of Broadly Neutralizing Antibodies in Humanized Mice. Nature (2012) 492(7427):118–22. 10.1038/nature11604 PMC3809838 23103874

[130] Lynch RM Boritz E Coates EE DeZure A Madden P Costner P. Virologic Effects of Broadly Neutralizing Antibody VRC01 Administration During Chronic HIV-1 Infection. Sci Transl Med (2015) 7(319):319ra206. 10.1126/scitranslmed.aad5752 PMC12366723 26702094

[131] Shingai M Donau OK Plishka RJ Buckler-White A Mascola JR Nabel GJ. Passive Transfer of Modest Titers of Potent and Broadly Neutralizing Anti-HIV Monoclonal Antibodies Block SHIV Infection in Macaques. J Exp Med (2014) 211(10):2061–74. 10.1084/jem.20132494 PMC4172223 25155019

[132] Shingai M Nishimura Y Klein F Mouquet H Donau OK Plishka R. Antibody-Mediated Immunotherapy of Macaques Chronically Infected With SHIV Suppresses Viraemia. Nature (2013) 503(7475):277–80. 10.1038/nature12746 PMC4133787 24172896

[133] Pegu A Yang ZY Boyington JC Wu L Ko SY Schmidt SD. Neutralizing Antibodies to HIV-1 Envelope Protect More Effectively *In Vivo* Than Those to the CD4 Receptor. Sci Transl Med (2014) 6(243):243ra288. 10.1126/scitranslmed.3008992 PMC4562469 24990883

[134] Julg B Tartaglia LJ Keele BF Wagh K Pegu A Sok D. Broadly Neutralizing Antibodies Targeting the HIV-1 Envelope V2 Apex Confer Protection Against a Clade C SHIV Challenge. Sci Transl Med (2017) 9(406):eaal1321. 10.1126/scitranslmed.aal1321 28878010 PMC5755978

[135] Ledgerwood JE Coates EE Yamshchikov G Saunders JG Holman L Enama ME. Safety, Pharmacokinetics and Neutralization of the Broadly Neutralizing HIV-1 Human Monoclonal Antibody VRC01 in Healthy Adults. Clin Exp Immunol (2015) 182(3):289–301. 10.1111/cei.12692 26332605 PMC4636891

[136] Mayer KH Seaton KE Huang Y Grunenberg N Isaacs A Allen M. Safety, Pharmacokinetics, and Immunological Activities of Multiple Intravenous or Subcutaneous Doses of an Anti-HIV Monoclonal Antibody, VRC01, Administered to HIV-Uninfected Adults: Results of a Phase 1 Randomized Trial. PloS Med (2017) 14(11):e1002435. 10.1371/journal.pmed.1002435 29136037 PMC5685476

[137] Morris L Mkhize NN. Prospects for Passive Immunity to Prevent HIV Infection. PloS Med (2017) 14(11):e1002436. 10.1371/journal.pmed.1002436 29136030 PMC5685477

[138] Caskey M Schoofs T Gruell H Settler A Karagounis T Kreider EF. Antibody 10-1074 Suppresses Viremia in HIV-1-Infected Individuals. Nat Med (2017) 23(2):185–91. 10.1038/nm.4268 PMC5467219 28092665

[139] Hua CK Ackerman ME. Increasing the Clinical Potential and Applications of Anti-HIV Antibodies. Front Immunol (2017) 8:1655. 10.3389/fimmu.2017.01655 29234320 PMC5712301

[140] Yang L Wang P. Passive Immunization Against HIV/AIDS by Antibody Gene Transfer. Viruses (2014) 6(2):428–47. 10.3390/v6020428 PMC3939464 24473340

[141] Keizer RJ Huitema AD Schellens JH Beijnen JH. Clinical Pharmacokinetics of Therapeutic Monoclonal Antibodies. Clin Pharm (2010) 49(8):493–507. 10.2165/11531280-000000000-00000 20608753

[142] Balazs AB Chen J Hong CM Rao DS Yang L Baltimore D. Antibody-Based Protection Against HIV Infection by Vectored Immunoprophylaxis. Nature (2011) 481(7379):81–4. 10.1038/nature10660 PMC3253190 22139420

[143] Balazs AB West AP Jr. Antibody Gene Transfer for HIV Immunoprophylaxis. Nat Immunol (2013) 14(1):1–5. 10.1038/ni.2480 23238748 PMC4560170

[144] Johnson PR Schnepp BC Zhang J Connell MJ Greene SM Yuste E. Vector-Mediated Gene Transfer Engenders Long-Lived Neutralizing Activity and Protection Against SIV Infection in Monkeys. Nat Med (2009) 15(8):901–6. 10.1038/nm.1967 PMC2723177 19448633

[145] Martinez-Navio JM Fuchs SP Pantry SN Lauer WA Duggan NN Keele BF. Adeno-Associated Virus Delivery of Anti-HIV Monoclonal Antibodies Can Drive Long-Term Virologic Suppression. Immunity (2019) 50(3):567–575 e565. 10.1016/j.immuni.2019.02.005 30850342 PMC6457122

[146] Priddy FH Lewis DJM Gelderblom HC Hassanin H Streatfield C LaBranche C. Adeno-Associated Virus Vectored Immunoprophylaxis to Prevent HIV in Healthy Adults: A Phase 1 Randomised Controlled Trial. Lancet HIV (2019) 6(4):e230-9. 10.1016/S2352-3018(19)30107-9 30885692 PMC6443625

[147] Haynes BF Fleming J St Clair EW Katinger H Stiegler G Kunert R. Cardiolipin Polyspecific Autoreactivity in Two Broadly Neutralizing HIV-1 Antibodies. Science (2005) 308(5730):1906–8. 10.1126/science.1111781 15860590

[148] Yang G Holl TM Liu Y Li Y Lu X Nicely NI. Identification of Autoantigens Recognized by the 2F5 and 4E10 Broadly Neutralizing HIV-1 Antibodies. J Exp Med (2013) 210(2):241–56. 10.1084/jem.20121977 PMC3570098 23359068

[149] Liu M Yang G Wiehe K Nicely NI Vandergrift NA Rountree W. Polyreactivity and Autoreactivity Among HIV-1 Antibodies. J Virol (2015) 89(1):784–98. 10.1128/JVI.02378-14 PMC4301171 25355869

[150] Gautam R Nishimura Y Gaughan N Gazumyan A Schoofs T Buckler-White A. A Single Injection of Crystallizable Fragment Domain-Modified Antibodies Elicits Durable Protection From SHIV Infection. Nat Med (2018) 24(5):610–6. 10.1038/s41591-018-0001-2 PMC5989326 29662199

[151] Ko SY Pegu A Rudicell RS Yang ZY Joyce MG Chen X. Enhanced Neonatal Fc Receptor Function Improves Protection Against Primate SHIV Infection. Nature (2014) 514(7524):642–5. 10.1038/nature13612 PMC4433741 25119033

[152] Saunders KO Pegu A Georgiev IS Zeng M Joyce MG Yang ZY. Sustained Delivery of a Broadly Neutralizing Antibody in Nonhuman Primates Confers Long-Term Protection Against Simian/Human Immunodeficiency Virus Infection. J Virol (2015) 89(11):5895–903. 10.1128/JVI.00210-15 PMC4442454 25787288

[153] Euler Z Bunnik EM Burger JA Boeser-Nunnink BD Grijsen ML Prins JM. Activity of Broadly Neutralizing Antibodies, Including PG9, PG16, and VRC01, Against Recently Transmitted Subtype B HIV-1 Variants From Early and Late in the Epidemic. J Virol (2011) 85(14):7236–45. 10.1128/JVI.00196-11 PMC3126573 21561918

[154] Davis-Gardner ME Alfant B Weber JA Gardner MR Farzan M. A Bispecific Antibody That Simultaneously Recognizes the V2-And V3-Glycan Epitopes of the HIV-1 Envelope Glycoprotein Is Broader and More Potent Than Its Parental Antibodies. Mbio (2020) 11(1):e03080–19. 10.1128/mBio.03080-19 PMC6960291 31937648

[155] Gardner MR Fetzer I Kattenhorn LM Davis-Gardner ME Zhou AS Alfant B. Anti-Drug Antibody Responses Impair Prophylaxis Mediated by AAV-Delivered HIV-1 Broadly Neutralizing Antibodies. Mol Ther (2019) 27(3):650–60. 10.1016/j.ymthe.2019.01.004 PMC6403482 30704961

[156] Bonsignori M Wiehe K Grimm SK Lynch R Yang G Kozink DM. An Autoreactive Antibody From an SLE/HIV-1 Individual Broadly Neutralizes HIV-1. J Clin Invest (2014) 124(4):1835–43. 10.1172/JCI73441 PMC3973118 24614107

[157] Hladik F Hope TJ. HIV Infection of the Genital Mucosa in Women. Curr HIV/AIDS Rep (2009) 6(1):20–8. 10.1007/s11904-009-0004-1 19149993

[158] Hladik F McElrath MJ. Setting the Stage: Host Invasion by HIV. Nat Rev Immunol (2008) 8(6):447–57. 10.1038/nri2302 PMC2587276 18469831

[159] Alvarez RA Barria MI Chen BK. Unique Features of HIV-1 Spread Through T Cell Virological Synapses. PloS Pathog (2014) 10(12):e1004513. 10.1371/journal.ppat.1004513 25522148 PMC4270788

[160] Jolly C Kashefi K Hollinshead M Sattentau QJ. HIV-1 Cell to Cell Transfer Across an Env-Induced, Actin-Dependent Synapse. J Exp Med (2004) 199(2):283–93. 10.1084/jem.20030648 PMC2211771 14734528

[161] Sagar M. HIV-1 Transmission Biology: Selection and Characteristics of Infecting Viruses. J Infect Dis (2010) 202:S289–96. 10.1086/655656 PMC2946383 20846035

[162] Moldt B Rakasz EG Schultz N Chan-Hui P-Y Swiderek K Weisgrau KL. Highly Potent HIV-Specific Antibody Neutralization *In Vitro* Translates Into Effective Protection Against Mucosal SHIV Challenge *In Vivo* . Proc Natl Acad Sci USA (2012) 109(46):18921–5. 10.1073/pnas.1214785109 PMC3503218 23100539

[163] Pegu A Yang Z-y Boyington JC Wu L Ko S-Y Schmidt SD. Neutralizing Antibodies to HIV-1 Envelope Protect More Effectively *In Vivo* Than Those to the CD4 Receptor. Sci Trans Med (2014) 6(243):243ra88. 10.1126/scitranslmed.3008992 PMC4562469 24990883

[164] Parsons MS Lloyd SB Lee WS Kristensen AB Amarasena T Center RJ. Partial Efficacy of a Broadly Neutralizing Antibody Against Cell-Associated SHIV Infection. Sci Trans Med (2017) 9(402):eaaf1483. 10.1126/scitranslmed.aaf1483 28794282

[165] Sigal A Kim JT Balazs AB Dekel E Mayo A Milo R. Cell-To-Cell Spread of HIV Permits Ongoing Replication Despite Antiretroviral Therapy. Nature (2011) 477(7362):95–U100. 10.1038/nature10347 21849975

[166] Liu Y Cao W Sun M Li T. Broadly Neutralizing Antibodies for HIV-1: Efficacies, Challenges and Opportunities. Emerging Microbes Infect (2020) 9(1):194–206. 10.1080/22221751.2020.1713707 PMC7040474 31985356

[167] Duncan CJA Williams JP Schiffner T Gaertner K Ochsenbauer C Kappes J. High-Multiplicity HIV-1 Infection and Neutralizing Antibody Evasion Mediated by the Macrophage-T Cell Virological Synapse. J Virol (2014) 88(4):2025–34. 10.1128/JVI.03245-13 PMC3911534 24307588

[168] Malbec M Porrot F Rua R Horwitz J Klein F Halper-Stromberg A. Broadly Neutralizing Antibodies That Inhibit HIV-1 Cell to Cell Transmission. J Exp Med (2013) 210(13):2813–21. 10.1084/jem.20131244 PMC3865481 24277152

[169] Reh L Magnus C Schanz M Weber J Uhr T Rusert P. Capacity of Broadly Neutralizing Antibodies to Inhibit HIV-1 Cell-Cell Transmission Is Strain- and Epitope-Dependent. PloS Pathog (2015) 11(7):e1004966. 10.1371/journal.ppat.1004966 26158270 PMC4497647

